# Mediterranean diet and endothelial function in patients with coronary heart disease: An analysis of the CORDIOPREV randomized controlled trial

**DOI:** 10.1371/journal.pmed.1003282

**Published:** 2020-09-09

**Authors:** Elena M. Yubero-Serrano, Carolina Fernandez-Gandara, Antonio Garcia-Rios, Oriol A. Rangel-Zuñiga, Francisco M. Gutierrez-Mariscal, Jose D. Torres-Peña, Carmen Marin, Javier Lopez-Moreno, Justo P. Castaño, Javier Delgado-Lista, Jose M. Ordovas, Pablo Perez-Martinez, Jose Lopez-Miranda

**Affiliations:** 1 Unidad de Gestión Clinica de Medicina Interna, Lipids and Atherosclerosis Unit, Maimonides Institute for Biomedical Research in Córdoba, Reina Sofia University Hospital, University of Córdoba, Córdoba, Spain; 2 CIBER Physiopathology of Obesity and Nutrition (CIBEROBN), Institute of Health Carlos III, Madrid, Spain; 3 Department of Cell Biology, Physiology and Immunology, University of Córdoba, IMIBIC/Reina Sofia University; Campus de Excelencia Internacional Agroalimentario (ceiA3), Córdoba, Spain; 4 Jean Mayer US Department of Agriculture Human Nutrition Research Center on Aging, Tufts University School of Medicine, Boston, Massachusetts, United States of America; University of Oxford, UNITED KINGDOM

## Abstract

**Background:**

Endothelial dysfunction is a crucial step in atherosclerosis development, and its severity is determinant for the risk of cardiovascular recurrence. Diet may be an effective strategy to protect the endothelium, although there is no consensus about the best dietary model. The CORonary Diet Intervention with Olive oil and cardiovascular PREVention (CORDIOPREV) study is an ongoing prospective, randomized, single-blind, controlled trial in 1,002 coronary heart disease (CHD) patients, whose primary objective is to compare the effect of 2 healthy dietary patterns (low-fat versus Mediterranean diet) on the incidence of cardiovascular events. Here, we report the results of one secondary outcome of the CORDIOPREV study: to evaluate the effect of these diets on endothelial function, assessed by flow-mediated dilation (FMD) of the brachial artery.

**Methods and findings:**

From the total participants taking part in the CORDIOPREV study, 805 completed endothelial function study at baseline and were randomized to follow a Mediterranean diet (35% fat, 22% monounsaturated fatty acids [MUFAs], and <50% carbohydrates) or a low-fat diet (28% fat, 12% MUFAs, and >55% carbohydrates), with endothelial function measurement repeated after 1 year. As secondary objectives and to explore different underlying mechanisms in the modulation of endothelial function, we quantified endothelial microparticles (EMPs) and endothelial progenitor cells (EPCs) and evaluated, in 24 preselected patients, in vitro cellular processes related to endothelial damage (reactive oxygen species, apoptosis, and senescence) and endothelial repair (cell proliferation and angiogenesis), as well as other modulators (micro-RNAs [miRNAs] and proteins). Patients who followed the Mediterranean diet had higher FMD (3.83%; 95% confidence interval [CI]: 2.91–4.23) compared with those in the low-fat diet (1.16%; 95% CI: 0.80 to 1.98) with a difference between diets of 2.63% (95% CI: 1.89–3.40, *p* = 0.011), even in those patients with severe endothelial dysfunction. We observed higher EPC levels (group difference: 1.64%; 95% CI: 0.79–2.13, *p* = 0.028) and lower EMPs (group difference: −755 EMPs/μl; 95% CI: −1,010 to −567, *p* = 0.015) after the Mediterranean diet compared with the low-fat diet in all patients. We also observed lower intracellular reactive oxygen species (ROS) production (group difference: 11.1; 95% CI: 2.5 to 19.6, *p* = 0.010), cellular apoptosis (group difference: −20.2; 95% CI: −26.7 to −5.11, *p* = 0.013) and senescence (18.0; 95% CI: 3.57 to 25.1, *p* = 0.031), and higher cellular proliferation (group difference: 11.3; 95% CI: 4.51 to 13.5, *p* = 0.011) and angiogenesis (total master segments length, group difference: 549; 95% CI: 110 to 670, *p* = 0.022) after the Mediterranean diet than the low-fat diet. Each dietary intervention was associated with distinct changes in the epigenetic and proteomic factors that modulate biological process associated with endothelial dysfunction. The evaluation of endothelial function is a substudy of the CORDIOPREV study. As in any substudy, these results should be treated with caution, such as the potential for false positives because of the exploratory nature of the analyses.

**Conclusions:**

Our results suggest that the Mediterranean diet better modulates endothelial function compared with a low-fat diet and is associated with a better balance of vascular homeostasis in CHD patients, even in those with severe endothelial dysfunction.

**Clinical trial registration:**

URL, http://www.cordioprev.es/index.php/en. clinicaltrials.gov number NCT00924937.

## Abstract 2

Jose Lopez-Miranda and colleagues compare endothelial function in patients with coronary heart disease who consumed different healthy diets.

## Author summary

**Why was this study done?:**

- Cardiovascular disease is the leading cause of morbidity and mortality in high- and middle-income countries and imposes a major economic burden on both the healthcare system and society.
- Endothelial dysfunction, a primary mechanism involved in the development of arteriosclerotic disease, is recognized as a significant predictor of cardiovascular risk. In fact, individuals with coronary heart disease (CHD) and endothelial dysfunction have a higher cardiovascular event recurrence rate compared with those with normal endothelial function.
- Considerable evidence supports the theory that lifestyle factors such as diet influence endothelial function. It is therefore a high priority to find therapeutic approaches that could improve endothelial dysfunction in the context of cardiovascular disease.

**What did the researchers do and find?:**

- We evaluated the effect of 2 healthy dietary models (a Mediterranean diet and a low-fat diet) on endothelial function, measured by flow-mediated dilation (FMD), in patients with CHD, as well as exploring the different biological underlying mechanisms.
- In a large group of patients with CHD (the CORonary Diet Intervention with Olive oil and cardiovascular PREVention [CORDIOPREV] study), those following the Mediterranean diet, compared with those on a low-fat diet, had higher FMD, even in patients with a severe endothelial dysfunction.
- The Mediterranean diet, compared with the low-fat diet, also led to better endothelial function, enhancing endothelial repair mechanisms and reducing the mechanisms associated with endothelial damage.

**What do these findings mean?:**

- Our results suggest that a Mediterranean diet pattern could be recommended as the best dietary strategy to recover endothelial dysfunction in patients with CHD.

## Introduction

Cardiovascular disease (CVD) remains the leading cause of morbidity and mortality in high- and middle-income countries [1, 2] and imposes a major economic burden on both the healthcare system and society. Endothelial dysfunction, a primary mechanism involved in the development of arteriosclerotic disease, is widely recognized as a significant predictor of cardiovascular risk [3, 4]. Over the last 2 decades, endothelial function has been evaluated by noninvasive methods, such as one that determines the endothelium-dependent dilation capacity of the brachial artery, flow-mediated dilation (FMD) [5]. Although the strong correlation between FMD and cardiovascular risk factors is well-established [6, 7], different studies have suggested that FMD may be more useful in screening for recurrent cardiovascular events in high-risk patients [8–10] than in a healthier general population [11]. Low FMD values have been related to a higher cardiovascular event rate and all-cause mortality than normal or high FMD values in the context of coronary heart disease (CHD) [12, 13].

In this context, increased levels of endothelial microparticles (EMPs; vesicles formed from endothelial cells membrane after injury) have been associated with endothelial injury and dysfunction [14]. Conversely, endothelial progenitor cells (EPCs) are related to the maintenance of endothelial integrity and function [15]. The functional properties of EPCs, including cell adherence, migration, invasion, and vessel formation, appear to be attenuated in patients with established CVD [16]. The EMPs/EPCs ratio is therefore becoming a marker of imbalance between endothelial injury and repair and may be related to the incidence of new cardiovascular events [17, 18].

Maintaining the correct endothelial function requires a balance between the mechanisms that protect its integrity and those that repair/recover from its dysfunction, known as vascular endothelial homeostasis [17]. This term refers to the balance in the production of reactive oxygen species (ROS), a decrease in the proapoptotic environment, and the ability to regenerate the endothelium through angiogenesis. These biological processes are regulated by an epigenetic component such as micro-RNAs (miRNAs) and exerted by specific proteins.

Strong evidence supports the idea that lifestyle factors, such as diet, influence endothelial function. While saturated fatty acid (SFA)-rich diets have been proven to impair endothelial function [19], the consumption of diets rich in monounsaturated fatty acids (MUFAs), such as the Mediterranean diet pattern, has been linked to improved endothelial function in metabolic syndrome [20, 21] as well as overweight/obesity or hypercholesterolemic patients [22–27]. A beneficial effect of substituting SFAs with MUFAs or n-6 polyunsaturated fatty acids (PUFAs) on circulating EPC and microparticle numbers has recently been described in individuals with moderate cardiovascular risk [19]. However, to the best of our knowledge, no studies have investigated the long-term dietary effect on endothelial function and mechanisms related to vascular endothelial homeostasis in a large-scale population of CHD patients. The importance of these studies lies in the fact that the cardiovascular event recurrence rate in CHD patients with endothelial dysfunction (low FMD levels) is higher compared with those with normal endothelial function [8]. It is therefore a high priority to find therapeutic approaches that could improve the endothelial dysfunction in this group of patients.

On the basis of the evidence discussed above, the primary objective of the CORonary Diet Intervention with Olive oil and cardiovascular PREVention (CORDIOPREV) study is to evaluate the efficacy of a Mediterranean diet, rich in MUFAs from olive oil, as compared with a low-fat diet to prevent clinical events and mortality in patients with previous CHD in a long-term follow-up study. The primary objective of this analysis was to evaluate the long-term effect of 2 healthy dietary models (Mediterranean diet and a low-fat diet) on restoring endothelial dysfunction in patients with CHD. A secondary analysis was to explore the biological process related to vascular endothelial homeostasis to identify a dietary model that recovers endothelial functionality and decreases the risk of recurrence of cardiovascular events in patients with CHD.

## Material and methods

### Study design

The current work was conducted within the framework of the CORDIOPREV study (clinicaltrials.gov number NCT00924937). The CORDIOPREV study is an ongoing prospective, randomized, single-blind, controlled trial including 1,002 CHD patients who had their last coronary event more than 6 months before enrollment. The primary objective of the CORDIOPREV study is to evaluate the efficacy of a Mediterranean diet, rich in MUFAs from olive oil, as compared with a low-fat diet to prevent clinical events and mortality in patients with previous CHD in a long-term follow-up study. The present study aims to evaluate a secondary endpoint of the CORDIOPREV study, the effect of the 2 dietary patterns on endothelial function (measured by FMD) [28].

Patients were recruited from November 2009 to February 2012, mostly at Reina Sofia University Hospital, Córdoba, Spain, but also from other hospitals in the neighboring provinces of Córdoba and Jaen. To summarize, patients were eligible if they were aged 20–75, with established CHD but without clinical events in the last 6 months, with the intention of following a long-term monitoring study, with no other serious illnesses and a life expectancy of at least 5 years. All patients gave their written informed consent to participate in the study. The study protocol was approved by the Human Investigation Review Committee at Reina Sofia University Hospital (consent orally obtained), according to institutional and Good Clinical Practice guidelines.

### Randomization and dietary intervention

Randomization was performed by the Andalusian School of Public Health, as previously described [28]. The study dietitians were the only members of the intervention team to know about the dietary group of each participant. Briefly, the randomization was based on the following variables: sex (male, female), age (<60 and ≥ 60 years old), and previous myocardial infarction (yes, no). Each patient was randomly stratified, in addition to the conventional treatment for CHD, to one of two potentially healthy diets: 1) the Mediterranean diet, with a minimum 35% of calories from fat (22% MUFAs, 6% PUFAs, and <10% SFAs), 15% proteins, and a maximum of 50% carbohydrates; and (2) a low-fat, high-complex carbohydrate diet, as recommended by the National Cholesterol Education Program (NCEP) and the American Heart Association (AHA), with <30% total fat (12%–14% MUFAs, 6%–8% PUFAs and <10% SFAs), 15% protein, and a minimum 55% carbohydrates. In both diets, the cholesterol content was adjusted to <300 mg/d. Full details on dietary assessment and follow-up visits, as well as biochemical measurements, are published elsewhere[28, 29]. The present study was performed in a follow-up period of 1 year. Details of the patients’ dietary adherence, energy, nutrients, and food intake (baseline and after 1 year of follow-up) are shown in S1 Text and S1, S2, S3 and S4 Tables.

In the Mediterranean diet group, the main recommendations were abundant use of virgin olive oil for cooking and dressing (≥4 tablespoons/day; 10–15 g/tablespoon); daily consumption of at least 2 servings of vegetables (200 g/serving; at least one serving raw or as salad) and 3 or more units of fresh fruit (125–150 g/unit); weekly consumption of at least 3 servings of legumes (150 g cooked weight/serving), 3 or more servings of fish or seafood (especially oily fish; 100–150 g/serving), and fresh nuts and seeds (3 or more handfuls per week); cooking dishes seasoned with “sofrito” (a slow-cooked homemade sauce with tomato, garlic, onion, aromatic herbs, and olive oil) at least twice a week; a reduction in meat consumption, choosing (skinless) white meat instead of red meat or processed meat (< 1 serving/day); and avoidance of additional fats (butter, margarine, seed oils, creams, etc.) and foods rich in sugar and unhealthy fats (commercial bakery products, chips, precooked food, sugared beverages, etc.).

In the low-fat diet group, participants received personalized recommendations according to the AHA and the NCEP dietary guidelines in use at the beginning of the study. The main recommendations were focused on limiting all types of fat consumption (both animal and vegetable) and on increasing the intake of complex carbohydrates. Specifically, they were advised to minimize the amount of oil used for cooking and dressing (≤2 tablespoons/day); not to eat more than 1 serving of red meat per week; choosing low-fat dairy products; consumption of lean fish instead of oily fish or fish/seafood canned in oil (≤1 serving/week); avoidance of nuts and seeds (≤1 serving/ week); to limit the consumption of commercial bakery goods, sweets, and pastries (≤ 1 serving/week); and to cook without the use of oil.

S4 Table provides the intake of main food groups at baseline and after 1 year of intervention. The consumption of whole grains (unrefined) instead of refined grains was recommended in both dietary intervention groups. Patients increased the intake of whole grains and reduced those refined grains after both the Mediterranean diet and the low-fat diet. Although the low-fat diet provided a greater intake of whole grains than the Mediterranean diet, the intake of vegetables, fruits, legumes, and nuts (good sources of fiber) increased in both diet groups, being more marked after the Mediterranean diet. In our study, although both dietary models share common characteristics in some of the major components (i.e., high intake of vegetables, fruit, legumes, and whole grains), patients consuming the Mediterranean diet also had a high intake of oily fish, nuts, and extra virgin olive oil, together with a low intake of harmful foods such as red/processed meats and pastries/commercial bakery products [29].

### Laboratory tests

The measurement of the anthropometric and biochemical parameters has been previously described [28].

### Study of the endothelial function

According to a predefined analysis of the CORDIOPREV study, we performed the evaluation of endothelial function by quantification of FMD of the brachial artery at the beginning of the study and after 1 year of dietary intervention, as previously described [30].

**Fig 1:**
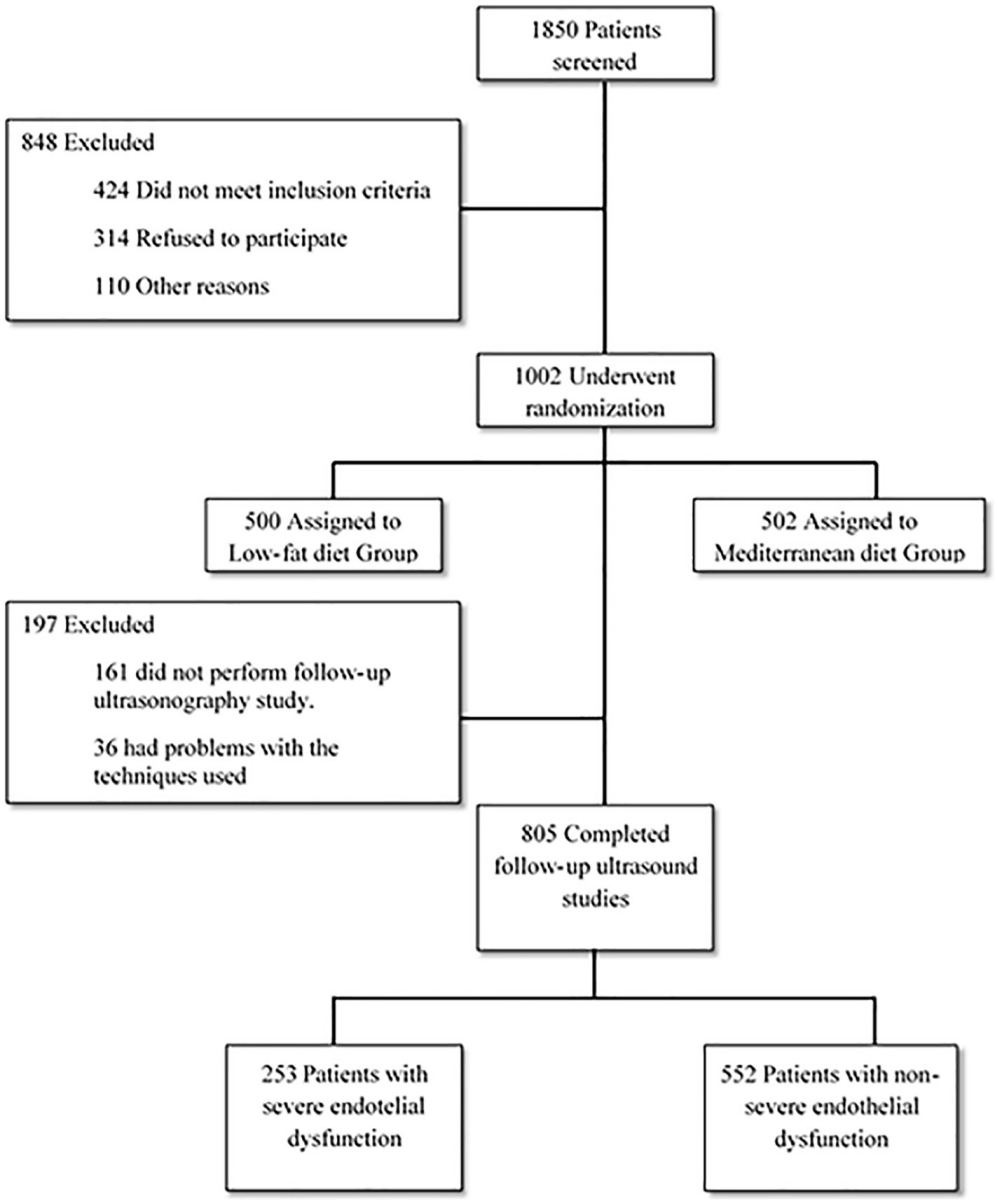
Screening and randomization flow-chart of the CORDIOPREV study and the ultrasound assessment of endothelial-dependent flow-mediated vasodilation of the brachial artery. CORDIOPREV, CORonary Diet Intervention with Olive oil and cardiovascular PREVention.

A total of 805 patients completed the ultrasonography study (n = 418, Mediterranean diet and n = 387, low-fat diet). The baseline characteristics of this subgroup of patients did not differ from the total CORDIOPREV cohort (n = 1,002) (S5 Table). 197 patients were excluded, mainly because they did not complete the ultrasonography study (at baseline or after 1 year of each diet: n = 161, 16%) or because of problems related to the technique (n = 36, 3.4%) (Fig 1).

### Endothelial dysfunction criteria

Patients were divided into tertiles according to their baseline FMD values: tertile 1 (<2%, low FMD), tertile 2 (2%–6.1%, medium FMD), and tertile 3 (≥6.1%, high FMD). In order to classify the patients according to the severity of the endothelial dysfunction, we used the FMD cutoff value of the first tertile (low values of FMD) to discriminate between those with severe endothelial dysfunction from the remaining patients with nonsevere endothelial dysfunction (tertiles 2 and 3). In this sense, we established 2 groups: patients with severe endothelial dysfunction (n = 253 with FMD < 2%; n = 132 for the Mediterranean diet, and n = 121 for the low-fat diet) and patients with nonsevere endothelial dysfunction (n = 552 with FMD ≥ 2%; n = 286 for the Mediterranean diet and n = 266 for the low-fat diet). Previous findings support that in the context of CHD, low values of FMD have been related more closely to a higher cardiovascular event rate and all-cause mortality than high FMD values [12, 13]. The estimation of the appropriate sample size used in the study is provided in S1 Text.

Other experiments and analyses (data-driven) were performed to elucidate the underlying mechanisms involved in the modulation of endothelial dysfunction (quantification of levels of EPCs and EMPs by flow cytometry and evaluation of intracellular ROS production and cellular apoptosis, senescence, proliferation, and angiogenesis by different in vitro approaches using 3 models of endothelial cells).

### Quantification of EPCs and EMPs

The quantification of the EPCs and EMPs was performed with flow cytometry in the total population (n = 805) before and after 1 year of dietary intervention. Details of the methodology used in these measurements are shown in S1 Text.

### Cell culture studies in endothelial-related cells

In order to determine the dietary effect on the underlying mechanisms involved in vascular endothelial homeostasis, we selected 24 patients: 12 patients with the lowest values of FMD and 12 patients with the highest values of FMD (with severe and nonsevere endothelial dysfunction, respectively). In both subgroups, the patients were distributed equally between the 2 diets (Mediterranean diet, n = 6 and low-fat diet, n = 6). The estimation of the appropriate sample size used in the study and a detailed description of these in vitro experiments are provided in S1 Text.

We used 2 models of endothelial cell cultures: human umbilical vein endothelial cells (HUVECs), as a well-established cellular model for endothelial function studies, to evaluate intracellular ROS production and cellular apoptosis (TUNEL assay) and EPCs derived and isolated from human umbilical venous blood from healthy female donors to measure cellular proliferation, senescence, and angiogenesis. However, because of the characteristics of the patients and in order to validate the in vitro experiments, we also used human coronary artery endothelial cells (HCAECs), which provide an excellent model for studying all the aspects of cardiovascular function and disease. A description of the characteristics of the endothelial cell and the cellular experiments is shown in S1 Text [31, 32].

In all the experiments, the cells were exposed to serum samples (10%) from each selected CHD patient (at baseline and 1 year after each dietary intervention), and each sample was analyzed in triplicate.

### Dietary effect on the biological process associated with endothelial dysfunction and vascular endothelial homeostasis: From epigenetic to biochemical and proteomic factors

In order to study the effect of the dietary intervention on biological mechanisms related to endothelial dysfunction and vascular endothelial homeostasis, we performed a full characterization of the serum samples from 24 select patients to perform the in vitro experiments. Thus, we studied the effect of diet on the epigenetic, biochemical, and proteomic factors that regulate the biological processes associated with endothelial dysfunction and vascular endothelial homeostasis: 1) serum levels of methylglyoxal (MG), a major precursor of the formation of advanced glycation end products (AGEs) involved in vascular endothelial damage; 2) serum proteome screening by SWATH-MS analysis; and 3) expression profile of serum miRNAs by next-generation sequencing. Details of the methodology used are shown in S1 Text.

### Statistical analysis

The statistical analyses were carried out using SPSS version 18.0 for Windows. The data are presented as the mean ± standard error for continuous variables. To evaluate the changes occurring in time, we calculated Δchanges (changes produced between post- and preintervention in each diet). We also evaluated group differences, defined as the differences in Δchanges in the Mediterranean group compared with Δchanges in the low-fat group, which included the 95% confidence interval (CI).

The biochemical variables were assessed for normal distribution, and skewed variables were normalized by log10 or square root transformation, as appropriate. In all cases, the data set was suitable for performing the statistical parametric test. FMD was categorized into 2 groups and analyzed as a categorical variable.

To evaluate the data variation, univariate and repeated-measures ANOVA analyses were used, as well as post hoc multiple comparisons analysis using the Bonferroni correction. Age, sex, and pharmacological treatments (beta blockers, angiotensin-receptor antagonist, angiotensin-converting enzyme inhibitors [ACEIs], nitrates, statins, and fibrates) were tested as covariates in all tests/assays. The differences were considered to be significant when *p* < 0.05.

Data from the quantified proteins (a total of 224 proteins) were transformed using the Perseus software suite 1.6.6.0 to achieve normal data distribution. The data were divided into 2 groups depending on FMD < 2% or FMD ≥ 2%, and a two-factor ANOVA was performed for diet and time. Statistical significances for the interaction between the 2 factors were identified with a *p*-value < 0.05 or −log(*p*-value) > 1.3 and were considered for hierarchical clustering and visualized by heatmap plots.

To analyze the expression profile of miRNAs, we performed a volcano plot analysis assessing the fold change of miRNAs with threshold >1.5 and *p*-value < 0.05 (−log10 of *p*-value = 1.3). Next, to validate the results obtained, we used the Reproducibility-Optimized Test Statistic (ROTS) package for R software (version 3.5.0) for group comparisons with a threshold of 0.05 for *p*-value [33]. The package analyzed the differences in groups with *t* statistics and provided the optimal selection of top-ranked miRNAs by using bootstrapping.

Based on the large number of miRNAs (2.083 miRNAs) studied, the objective of the analysis was to identify those miRNAs that showed significant differences after the intervention period with the 2 dietary models, both in patients with FMD ≥ 2 and in those with FMD < 2.

## Results

### Baseline characteristics of the study population

Baseline demographic and metabolic characteristics of the CHD patients according to the randomized dietary pattern and based on the severity of endothelial dysfunction are presented in Table 1.

**Table 1:** Baseline characteristics of the total CHD patients and classified according to the severity of endothelial dysfunction^1^^,^^2^.

	Low-Fat Diet (n = 387)			Mediterranean Diet (n = 418)		
	Total population	FMD < 2% (n = 121)	FMD ≥ 2% (n = 266)	Total population	FMD < 2% (n = 132)	FMD ≥ 2% (n = 286)
Age (years)	59.9 ± 0.5	60.1 ± 0.8	59.7 ± 0.5	60.4 ± 0.5	60.8 ± 0.8	60.0 ± 0.5
Men/Women	364/23	110/11	254/12	380/38	116/16	264/22
Weight	85.5 ± 0.7	86.4 ± 0.7	84.6 ± 0.8	84.7 ± 0.7	84.7 ± 1.2	84.6 ± 0.8
BMI (kg/m^2^)	31.2 ± 0.2	31.7 ± 0.5	30.8 ± 0.3	30.9 ± 0.2	31.1 ± 0.1	30.8 ± 0.2
Waist circumference (mm)	105.2 ± 0.6	105.9 ± 0.9	104.5 ± 0.7	104.9 ± 0.6	105.6 ± 0.9	104.2 ± 0.7
DBP (mmHg)	76.6 ± 0.5	76.0 ± 0.9	77.2 ± 0.7	77.2 ± 0.5	76.9 ± 0.5	77.4 ± 0.7
SBP (mmHg)	134.1 ± 1.0	135.4 ± 6.0	132.6 ± 4.1	137.1 ± 0.9	137.9 ± 0.9	136.2 ± 1.1
LDL cholesterol (mg/dL)	90.2 ± 1.3	94.4 ± 1.3	86.1 ± 1.6	89.9 ± 1.3	92.4 ± 2.4	87.3 ± 1.5
HDL cholesterol (mg/dL)	42.0 ± 0.5	42.4 ± 0.9	41.5 ± 0.5	41.8 ± 0.5	41.9 ± 0.9	41.6 ± 0.6
Total cholesterol (mg/dL)	158.9 ± 1.6	161.6 ± 1.6	155.6 ± 1.8	157.4 ± 1.5	159.2 ± 2.9	155.8 ± 1.8
Triglycerides (mg/dL)	135.8 ± 3.3	134.9 ± 6.6	136.2 ± 4.2	131.1 ± 3.2	130.9 ± 6.0	131.6 ± 3.5
Fasting glucose (mg/dL)	119.2 ± 1.8	121.6 ± 5.3	116.5 ± 2.9	118.3 ± 1.7	120.8 ± 4.7	116.9 ± 2.7
Fasting insulin (mU/L)	11.9 ± 0.6	12.9 ± 2.1	10.9 ± 1.8	11.6 ± 0.5	11.9 ± 1.9	11.5 ± 0.9
HbA1c (%)	6.59 ± 0.06	6.74 ± 0.04	6.52 ± 0.06	6.55 ± 0.05	6.66 ± 0.04	6.51 ± 0.03
hsCRP (mg/mL)	3.11 ± 0.18	3.30 ± 0.53	2.90 ± 0.30	3.04 ± 0.17	3.41 ± 0.58	2.71 ± 0.30
FMD (%)	3.01 ± 0.30	-1.32 ± 0.44	4.21 ± 0.33	4.06 ± 0.29	-2.89 ± 0.42	4.15 ± 0.35
Circulating EPCs (%)	3.41 ± 0.29	2.61 ± 0.29	4.21 ± 0.28	3.22 ± 0.31	2.43 ± 0.31	3.99 ± 0.34
EMP number/μL	2,995 ± 89	3,440 ± 91	2,648 ± 89	2,978 ± 101	3,508 ± 63	2,440 ± 57
EMP/EPC ratio	3.10 ± 0.38	3.61 ± 0.38	2.41 ± 0.29	3.43 ± 0.40	3.97 ± 0.30	2.69 ± 0.20
Alcohol drinkers (%)	53.9	55.2	51.1	56.0	56.0	57.4
Smoking (%)	10.1	9.34	10.91	10.6	10.11	11.04
**Antihypertensive drugs (%)**						
ACEIs or ARBs	46.0	46.3	45.9	40.8	42.4	38.5
Beta blockers	66.1	62.8	67.7	59.5	61.4	58.7
Nitrates	10.3	9.1	10.9	6.7	4.5	7.6
**Lipid lowering drugs (%)**						
Statins	86.6	86.8	86.5	84.5	81.8	85.8
Fibrates	1.3	0.8	1.5	1.9	2.3	1.7

^1^Values are represented as the mean ± standard error. Total population number; low-fat diet versus Mediterranean diet, *p* < 0.05 (univariate ANOVA).

^2^FMD < 2%, severe endothelial dysfunction; FMD ≥ 2%, nonsevere endothelial dysfunction.

**Abbreviations:** ACEI, angiotensin-converting enzyme inhibitor; ARB, angiotensin-receptor blocker; BMI, body mass index; CHD, coronary heart disease; DBP, diastolic blood pressure; EMP, endothelial microparticle; EPC, endothelial progenitor cell; FMD, flow-mediated dilation of the brachial artery; HbA1c, glycated hemoglobin; HDL, high-density lipoprotein; hsCRP, high sensitive C-reactive protein; LDL, low-density lipoprotein; SBP, systolic blood pressure.

### Effect of dietary intake on anthropometric and biochemical markers

The effect of each dietary intervention (Δchanges produced between post- and preintervention) on anthropometric and biochemical parameters in the total population and classified according to the severity of the endothelial dysfunction are presented in Tables 2 and 3, respectively. We also showed the group differences, defined as the differences in Δchanges in the Mediterranean group compared to Δchanges in the low-fat group (95% CI).

In the total population, we observed higher HDL cholesterol levels (0.76 mg/dL; 95% CI: 0.11 to 1.04, *p* = 0.020) and lower fasting glucose (−0.96 mg/dL;95% CI: −1.31 to −0.56, *p* = 0.011) and hsCRP levels (−0.44 mg/mL; 95% CI: −0.79 to −0.08, *p* = 0.009) in those patients who consumed the Mediterranean diet compared with those who consumed the low-fat diet (Table 2).

**Table 2:** Effect of dietary models on anthropometric and biochemical markers in CHD patients^1^.

	Low-Fat Diet (n = 387)	Mediterranean Diet (n = 418)	Group Difference^2^ (95% CI)	*p*-Value*
BMI (kg/m^2^)	−0.60 ± 0.05	−0.76 ± 0.08	−0.16 (−0.29 to 0.11)	0.621
LDL cholesterol (mg/dL)	−0.17 ± 0.11	−0.21 ± 0.09	−0.04 (−0.11 to 0.08)	0.803
HDL cholesterol (mg/dL)	−0.60 ± 0.20	0.16 ± 0.05	0.76 (0.11 to 1.04)	0.020
Total cholesterol (mg/dL)	1.48 ± 0.52	1.35 ± 0.19	−0.13 (−0.25 to 0.03)	0.785
Triglycerides (mg/dL)	−6.85 ± 0.21	−6.80 ± 0.53	0.05 (−0.08 to 0.14)	0.832
Fasting glucose (mg/dL)	−1.21 ± 0.37	−2.18 ± 0.15	−0.96 (−1.31 to −0.56)	0.011
Fasting insulin (mU/L)	−1.98 ± 0.12	−2.83 ± 0.81	−0.85 (−0.74 to 0.18)	0.090
HbA1c (%)	−0.07 ± 0.01	−0.09 ± 0.01	−0.02 (−0.19 to 0.10)	0.951
hsCRP (mg/mL)	0.11 ± 0.03	−0.55 ± 0.01	−0.44 (−0.79 to −0.08)	0.009

^1^Values are represented as the mean (Δchanges produced between post- and preintervention) ± standard error.

^2^Group difference: *p < 0.05, differences in Δchanges in the Mediterranean group compared with Δchanges in the low-fat group. **Abbreviations:** BMI, body mass index; CHD, coronary heart disease; CI, confidence interval; HbA1c, glycated hemoglobin; HDL, high-density lipoprotein; hsCRP, high sensitive C-reactive protein; LDL, low-density lipoprotein.

**Table 3:** Effect of dietary intervention on anthropometric and biochemical markers (Δchanges produced between post- and preintervention) in CHD patients classified according to the severity of endothelial dysfunction^1^.

		Severe Endothelial Dysfunction, FMD < 2% (n = 253)			Nonsevere Endothelial Dysfunction, FMD ≥ 2% (n = 552)			
	Low-fat diet (n = 121)	Mediterranean diet (n = 132)	Group difference^2^ (95% CI)	*p*-value*	Low-fat diet (n = 266)	Mediterranean diet (n = 286)	Group difference^*2*^ (95% CI)	*p*-value*
BMI (kg/m^2^)	−0.61 ± 0.03	−1.02 ± 0.07	−0.41 (−0.59 to 0.13)	0.089	−0.60 ± 0.07	−0.51 ± 0.02	0.09 (−0.19 to 0.25)	0.891
LDL cholesterol (mg/dL)	−2.10 ± 0.31	−2.82 ± 0.31	−0.72 (−0.91 to 0.38)	0.333	1.76 ± 0.40	2.41 ± 0.40	0.65 (−0.11 to 0.98)	0.190
HDL cholesterol (mg/dL)	−0.31 ± 0.20	−0.10 ± 0.05	0.21 (−0.31 to 0.32)	0.522	−0.30 ± 0.02	0.42 ± 0.06	0.72 (0.21 to 0.92)	0.026
Total cholesterol (mg/dL)	1.63 ± 0.12	0.69 ± 0.09	−0.94 (−1.25 to 0.63)	0.103	1.32 ± 0.91	2.02 ± 0.89	0.70 (−0.25 to 1.03)	0.217
Triglycerides (mg/dL)	0.49 ± 0.02	−4.50 ± 0.22	−4.99 (−5.88 to −3.14)	0.036	−14.2 ± 2.1	−9.10 ± 1.50	5.10 (−20.1 to 10.1)	0.541
Fasting glucose (mg/dL)	−0.82 ± 0.03	−1.05 ± 0.09	−0.23 (−0.41 to 0.01)	0.678	−1.61 ± 0.32	−3.31 ± 0.21	−1.70 (−2.01 to −1.56)	0.040
Fasting insulin (mU/L)	−2.15 ± 0.12	−2.76 ± 0.81	−0.61 (−1.04 to 0.18)	0.781	−1.82 ± 0.93	−2.91 ± 0.65	−1.09 (−1.34 to 0.68)	0.588
HbA1c (%)	−0.10 ± 0.02	−0.03 ± 0.01	0.07 (−0.17 to 0.30)	0.905	−0.04 ± 0.02	−0.14 ± 0.03	−0.01 (−0.19 to 0.15)	0.813
hsCRP (mg/mL)	0.14 ± 0.03	−0.19 ± 0.01	−0.05 (−0.30 to 0.03)	0.219	0.09 ± 0.08	−0.61 ± 0.03	−0.70 (−0.84 to −0.18)	0.035

^1^FMD of the brachial artery < 2%: severe endothelial dysfunction; ≥2%: nonsevere dysfunction. Values are represented as the mean (Δchanges produced between post- and preintervention) ± standard error.

^2^Group difference: *p < 0.05, differences in Δchanges in the Mediterranean group compared with Δchanges in the low-fat group. **Abbreviations:** BMI, body mass index; CHD, coronary heart disease; CI, confidence interval; FMD, flow-mediated dilation; HbA1c, glycated hemoglobin; HDL, high-density lipoprotein; hsCRP, high sensitive C-reactive protein; LDL, low-density lipoprotein.

### Mediterranean diet increased FMD regardless of the severity of the endothelial dysfunction

In the total population, we observed an increase in FMD after consumption of the Mediterranean diet compared to baseline (*p* = 0.001). The low-fat diet did not exert any effect on FMD.

**Fig 2:**
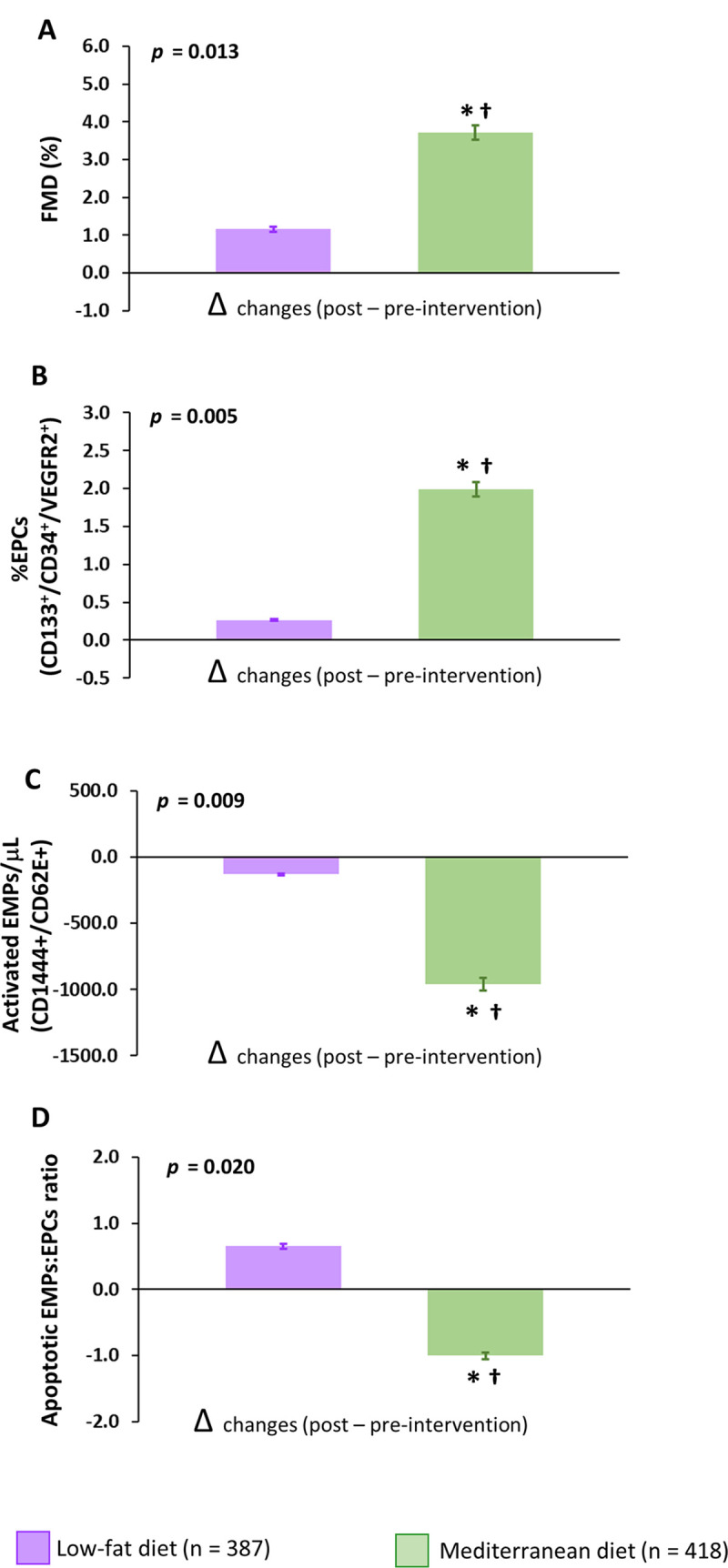
Effect of dietary intervention on endothelial function and other parameters related to endothelial functionality in total patients. (A) FMD, (B) circulating EPCs, (C) EMPs, and (D) EMP/EPC ratio. Data are presented as Δchanges produced between post- and preintervention ±SE. Variables were compared using the analysis of variance (univariate ANOVA), adjusted by age, sex, and pharmacological treatment. *Significant changes between Mediterranean diet and low-fat diet (*p* < 0.05). †Significant changes between post- and preintervention in each diet (*p* < 0.05). CD, cluster of differentiation; EPC, endothelial progenitor cell; EMP, endothelial microparticle; FMD, flow-mediated dilation; VEGFR2, Vascular Endothelial Growth Factor Receptor 2.

**Fig 3:**
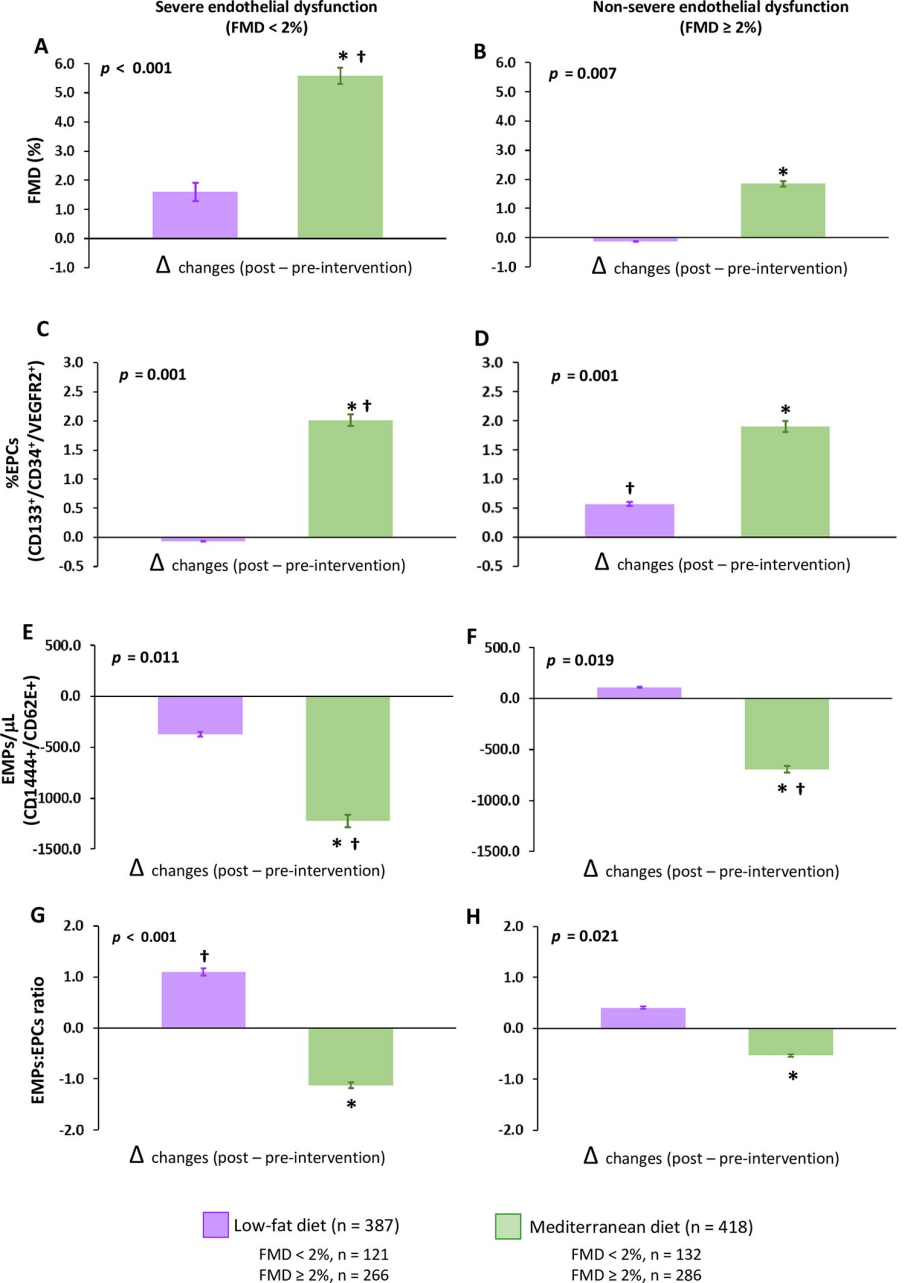
Effect of dietary intervention on endothelial function and other parameters related to endothelial functionality in patients classified according to the severity of endothelial dysfunction. (A and B) FMD, (C and D) circulating EPCs, (E and F) EMPs, and (G and H) EMP/EPC ratio. FMD < 2%, patients with severe endothelial dysfunction; FMD ≥ 2%, patients with nonsevere endothelial dysfunction. Data are presented as Δchanges produced between post- and preintervention ±SE. Variables were compared using the analysis of variance (univariate ANOVA), adjusted by age, sex, and pharmacological treatment. *Significant changes between Mediterranean diet and low-fat diet (*p* < 0.05). †Significant changes between post- and preintervention in each diet (*p* < 0.05). CD, cluster of differentiation; EPC, endothelial progenitor cell; EMP, endothelial microparticle; FMD, flow-mediated dilation; VEGFR2, Vascular Endothelial Growth Factor Receptor 2.

When comparing both dietary arms, we observed higher FMD after consumption of the Mediterranean diet compared with the low-fat diet (group difference of 2.63%; 95% CI: 1.89–3.40, *p* = 0.011) (Fig 2A). These findings were also reproduced when we stratified the population into those with severe endothelial dysfunction (group difference of 3.72%; 95% CI: 2.22–4.02, *p* = 0.006) and nonsevere endothelial dysfunction (group difference of 2.09%; 95% CI: 1.63–2.82, *p* = 0.019) (Fig 3A and 3B**)**.

### Mediterranean diet modulated in vivo markers of endothelial repair mechanisms and endothelial damage

In the total population, patients who consumed the Mediterranean diet, but not those who consumed the low-fat diet, increased EPC levels compared with baseline (*p* = 0.002). Moreover, we observed higher EPC levels after consumption of the Mediterranean diet compared with the low-fat diet (group difference of 1.64%; 95% CI: 0.79–2.13, *p* = 0.028) (Fig 2B). The Mediterranean diet was also associated with lower EMP levels compared with the low-fat diet (group difference of −755 EMPs/μl; 95% CI: −1,010 to −567, *p* = 0.015) and with improved EMP/EPC ratio (group difference of −0.16; 95% CI: −0.29 to −0.14, *p* = 0.032) (Figs 1D and 2C).

According to the severity of the endothelial dysfunction, we also observed higher EPC levels after consumption of the Mediterranean diet compared with the low-fat diet in patients with severe endothelial dysfunction (group difference of 2.06%; 95% CI: 1.72 to 3.02, *p* = 0.006) and nonsevere endothelial dysfunction (group difference of 1.40%; 95% CI: 1.13 to 2.03, *p* = 0.019) (Fig 3C and 3D). Patients following the Mediterranean diet had lower EMP levels compared with those following the low-fat diet (−801 EMPs/ml; 95% CI: −1,231 to −662, *p* = 0.005 and −850 EMPs/ml; 95% CI: −910 to −501, *p* = 0.012) in patients with severe and nonsevere endothelial dysfunction, respectively, and an improved EMP/EPC ratio (−1.93; 95% CI: −2.19 to −0.35, *p* = 0.016 and −1.22; 95% CI: −1.57 to −0.61, *p* = 0.023) in patients with severe and nonsevere endothelial dysfunction, respectively (Fig 3E–3H). After the low-fat diet, there was also a slight increase in EPC concentration in patients with nonsevere endothelial dysfunction compared with baseline (Fig 3D). However, patients with severe endothelial dysfunction showed an increase in EMP/EPC ratio but did not show any effect on EPC levels (Fig 3G and 3C, respectively). A summary of these results is shown in S1 Fig.

### Effect of dietary intervention on the biological process associated with endothelial dysfunction and vascular endothelial homeostasis: In vitro approaches

To assess the effect of the diet on the involved mechanisms in maintaining the integrity of the endothelium and leading to an improvement of endothelial dysfunction, we performed in vitro assays using HUVEC, EPC, and HCAEC cultures as endothelial cell models. All these models of cells were exposed to serum samples from 24 selected patients of the study (12 patients who consumed a Mediterranean diet, 6 with nonsevere and 6 with severe endothelial dysfunction, and 12 who consumed a low-fat diet, 6 with nonsevere and 6 with severe endothelial dysfunction). We also performed a characterization of the composition of these serum samples in order to evaluate the potential modulators that could be involved in the mechanisms related to vascular endothelial homeostasis (see Material and Methods section for more details). Baseline characteristics of these selected patients are shown in S6 Table.

#### Dietary effect on the cellular processes related to endothelial damage

**Fig 4:**
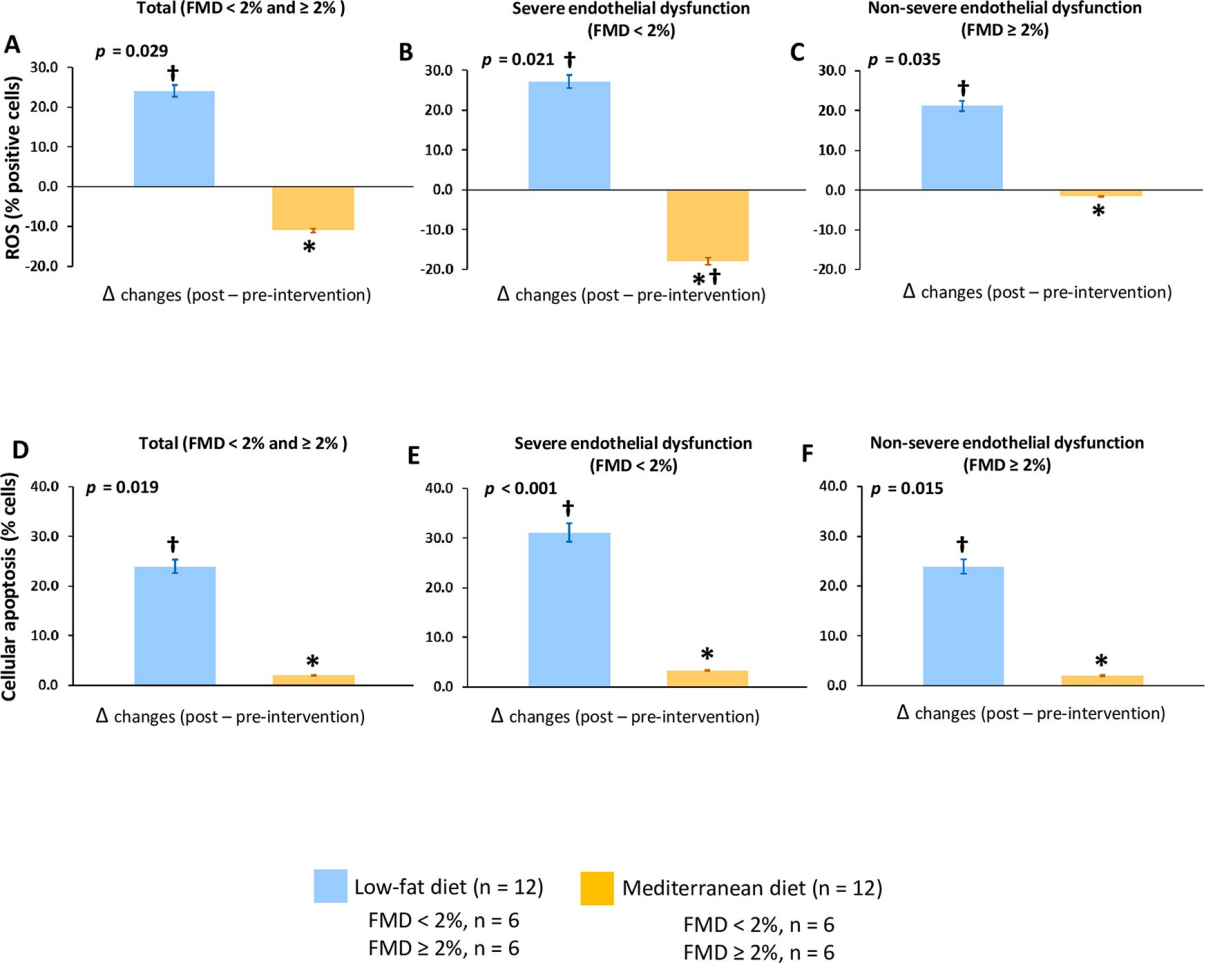
Effect of dietary intervention on in vitro intracellular ROS production and cellular apoptosis. (A, B, and C) Intracellular ROS production in total patients and classified according severity of endothelial dysfunction, respectively. (D, E, and F) Cellular apoptosis in total patients and classified according severity of endothelial dysfunction, respectively. All in vitro experiments were performed in HUVECs incubated with serum samples from 24 selected CHD patients. FMD < 2%, patients with severe endothelial dysfunction; FMD ≥ 2%, patients with nonsevere endothelial dysfunction. Data are presented as Δchanges produced between post- and preintervention ±SE. Variables were compared using the analysis of variance (univariate ANOVA). *Significant changes between Mediterranean diet and low-fat diet (*p* < 0.05). †Significant changes between post- and preintervention in each diet (*p* < 0.05). CHD, coronary heart disease; FMD, flow-mediated dilation; HUVEC, human umbilical vein endothelial cell; ROS, reactive oxygen species.

**Fig 5:**
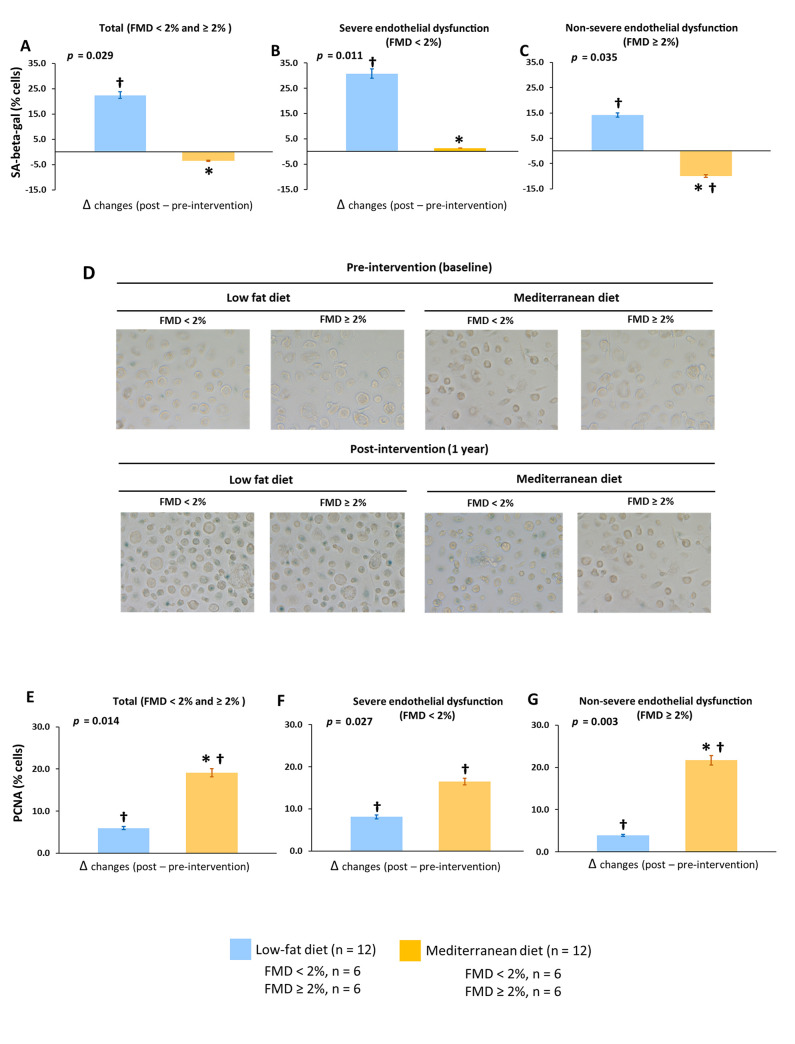
Effect of dietary intervention on in vitro cellular senescence and cellular proliferation. **5.** (A, B, and C) Cellular senescence in total patients and classified according severity of endothelial dysfunction, respectively. (E, F, and G) Cellular proliferation in total patients and classified according severity of endothelial dysfunction, respectively. (D) Representative optical microscopy images of the in vitro senescence assay at the final time point (24 h) (40×). All in vitro experiments were performed in EPCs incubated with serum samples from 24 selected CHD patients. FMD < 2%, patients with severe endothelial dysfunction; FMD ≥ 2%, patients with nonsevere endothelial dysfunction. Data are presented as Δchanges produced between post- and preintervention ±SE. Variables were compared using the analysis of variance (univariate ANOVA). †Significant changes between post- and preintervention in each diet (*p* < 0.05). *Significant changes between Mediterranean diet and low-fat diet (*p* < 0.05). CHD, coronary heart disease; EPC, endothelial progenitor cell; FMD, flow-mediated dilation; PCNA, proliferating cell nuclear antigen; SA-beta-gal, senescence-associated beta-galactosidase.

Serum from patients who consumed a low-fat diet increased intracellular ROS production and cellular apoptosis in HUVECs and HCAECs compared with baseline regardless of the severity of the endothelial dysfunction (all *p* < 0.05) (Fig 4A–4F and S2A–S2F Fig). Moreover, we observed increased cellular senescence in both EPCs and HCAECs with serum from patients who consumed this diet compared with baseline (all *p* < 0.05) (Fig 5A–5D and S3A–S3D Fig). However, serum from patients consuming the Mediterranean diet produced lower intracellular ROS production (group difference of 11.1; 95% CI: 2.5 to 19.6, *p* = 0.010) and cellular apoptosis (group difference of −20.2; 95% CI: −26.7 to −5.11, *p* = 0.013) and senescence (18.0; 95% CI: 3.57 to 25.1, *p* = 0.031) in all patients and also regarding the severity of endothelial dysfunction compared with the low-fat diet.

#### Dietary effect on the cellular endothelial regenerative capacity

**Fig 6:**
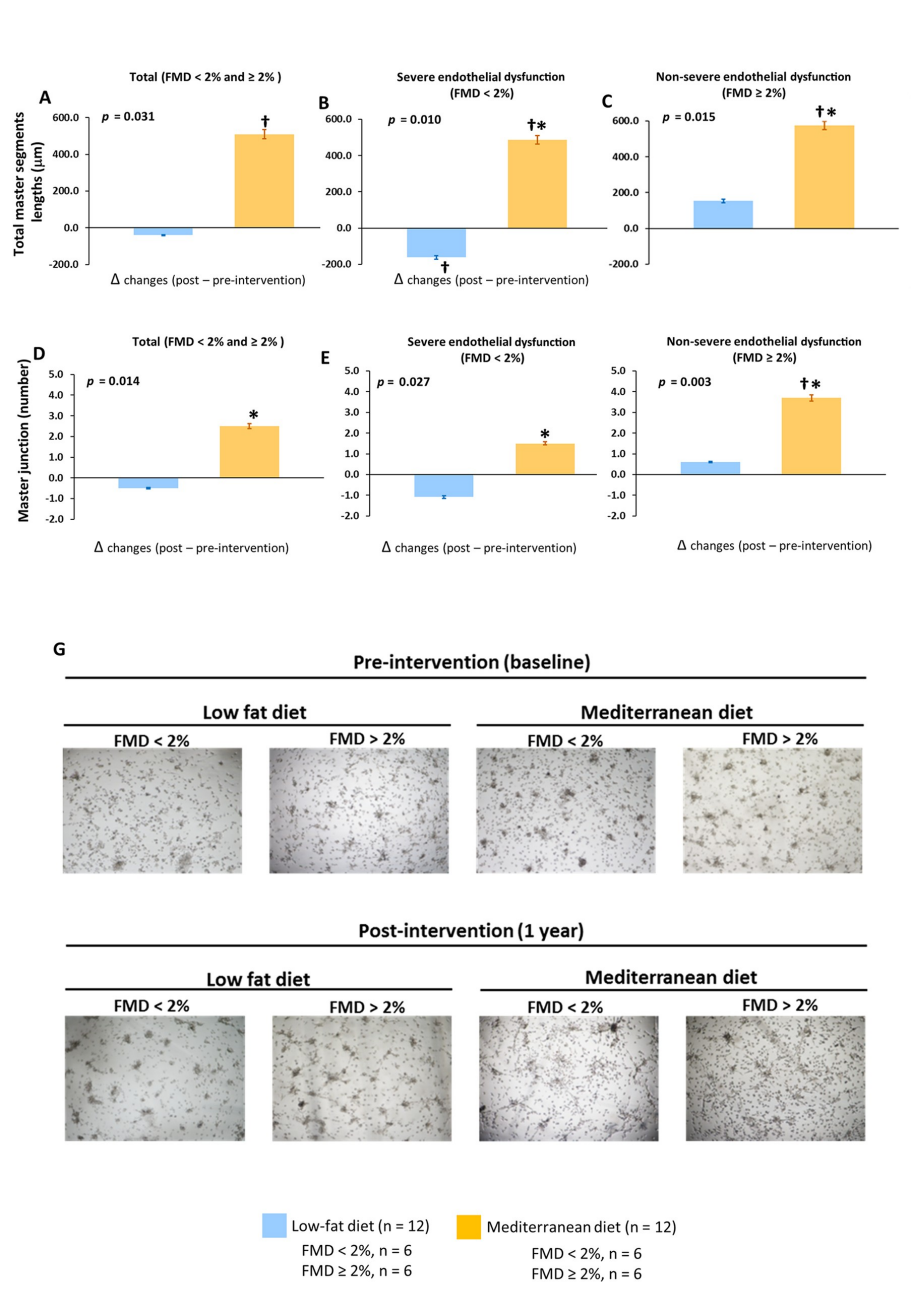
Effect of dietary intervention on in vitro angiogenic capacity. (A, B, and C) Total master segment length in total patients and classified according severity of endothelial dysfunction, respectively. (D, E, and F) Number of master junctions in total patients and classified according severity of endothelial dysfunction, respectively. (G) Representative optical microscopy images of the formation of vessels in the in vitro angiogenesis assay on Matrigel at the final time point (10 h) (40×). All in vitro experiments were performed in EPCs incubated with serum samples from 24 selected CHD patients. FMD < 2%, patients with severe endothelial dysfunction; FMD ≥ 2%, patients with nonsevere endothelial dysfunction. Data are presented as Δchanges produced between post- and preintervention ±SE. Variables were compared using the analysis of variance (univariate ANOVA). †Significant changes between post- and preintervention in each diet (*p* < 0.05). *Significant between Mediterranean diet and low-fat diet (*p* < 0.05). CHD, coronary heart disease; EPCs, endothelial progenitor cells; FMD, flow-mediated dilation.

Serum from patients who consumed a Mediterranean diet, but not the low-fat diet, increased cellular proliferation in both EPCs and HCAECs (Fig 5E–5G and S3E–S3G Fig) and angiogenesis in EPCs (total master segments length and master junction number) compared with baseline, regardless of the severity of the endothelial dysfunction of the patients (all *p* < 0.05) (Fig 6A–6G). Moreover, we observed a higher cellular proliferation (group difference of 11.3; 95% CI: 4.51–13.5, *p* = 0.011) and angiogenesis (total master segments length, group difference of 549; 95% CI: 110–670, *p* = 0.022; master junction number, group difference of 3.64; 95% CI: 1.76–5.70, *p* = 0.029) after consumption of the Mediterranean diet compared with the low-fat diet in all patients and also according to the severity of endothelial dysfunction.

#### MG levels determined an increase in ROS production and in the antiangiogenic capacity of the serum

Serum from patients who consumed a Mediterranean diet contained lower levels of MG (group difference of 0.58; 95% CI: 0.23–1.05, *p* = 0.025) compared with those who consumed a low-fat diet. This difference remains constant regardless of the severity of the endothelial dysfunction (all *p* < 0.05). Moreover, we observed an increase in MG levels after the low-fat diet compared to baseline in patients both with and without severe endothelial dysfunction (S4 Fig).

MG levels (changes produced between post- and preintervention) correlated positively with in vitro ROS production (*r* = 0.685, *p* = 0.006) and negatively with in vitro angiogenesis capacity (total master segment length) (*r* = −0.532, *p* = 0.012).

#### Serum proteome of patients with severe and nonsevere endothelial dysfunction is modulated by diet

**Fig 7:**
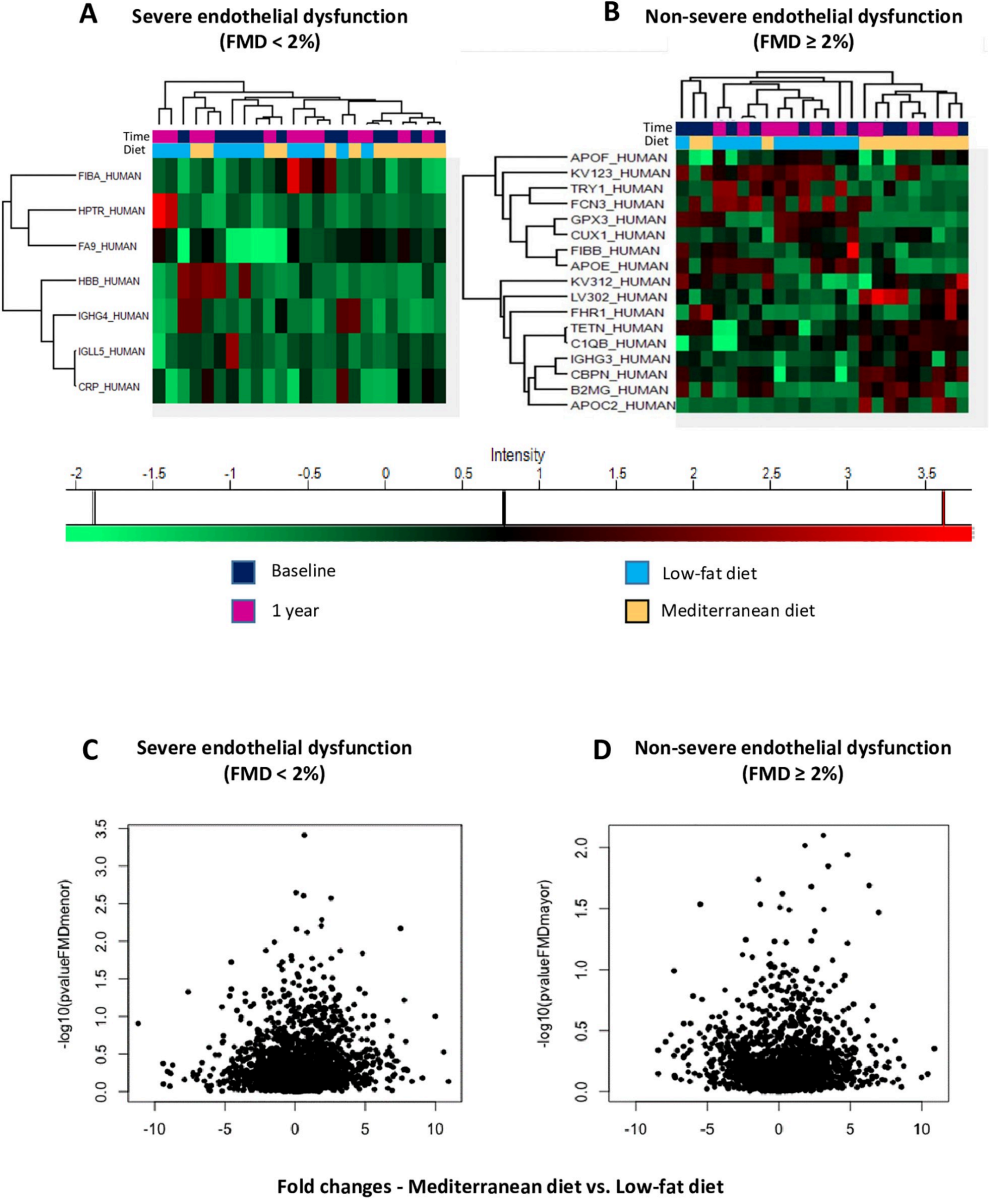
Protein and miRNAs pattern differentially expressed in serum samples according to time and diet interaction. (A) Heatmap of differentially expressed serum proteins in patients with severe endothelial dysfunction. (B) Heatmap of differentially expressed serum proteins in patients with nonsevere endothelial dysfunction. Eight and nine proteins were up- and down-regulated, respectively, after consumption of the low-fat diet, and inversely after consumption of the Mediterranean diet. (C) Volcano plots to assess significant changes of miRNAs expression after 1 year of each dietary intervention and according to FMD <2%; D) Volcano plots to assess significant changes of miRNAs expression after 1 year of each dietary intervention and according to FMD ≥ 2%. The selection criteria of miRNAs with a fold change greater than 1.5 and *p*-value > 0.05 (−log10(*p*-value) = 1.3). Analysis was performed with software R using the ROTS package (version 3.5.0) and R studio (version 1.1.442). APOC2, Apolipoprotein C-II; APOE, Apolipoprotein E; APOF, Apolipoprotein F; B2MG, β-2-microglobin; CBPN, Carboxipeptidase N catalytic chain; CRP, C-reactive protein; CUX1, Homeobox protein cut-like 1; C1QB, Complement C1q subcomponent subunit β; FA9, Coagulation factor IX; FCN3, Ficolin 3; FHR1, complement factor H-related protein; FIBA, Fibrinogen α chain; FIBB, Fibrinogen β chain; FMD, flow-mediated dilation; GPx3, glutathione peroxidase 3; HBB, Hemoglobin subunit β; HPTR, Haptoglobin-related protein; IGHG3, Ig γ-3 chain C region; IGHG4, Ig γ 4 chain; IGLL5, Immunoglobin λ like peptide 5; KV123, Ig κ chain V-I region; KV312, Ig κ chain V-III region; LV302, Ig λ chain V-III region; miRNA, XXX; ROTS, Reproducibility-Optimized Test Statistic; TETN, Tetranectin; TRY1, Trypsin 1.

A total of 224 proteins were identified in all the in vitro serum samples from 24 selected CHD patients. In patients with severe endothelial dysfunction, fibrinogen α chain, haptoglobin-related protein, and coagulation factor IX were up-regulated after consumption of the low-fat diet, whereas hemoglobin subunit β and C-reactive protein (CRP) were down-regulated after consumption of the Mediterranean diet (Fig 7A). In patients with nonsevere endothelial dysfunction, a first cluster of proteins (apolipoprotein F and E, glutathione peroxidase 3 [GPx3], and fibrinogen β chain) was up-regulated and down-regulated after the low-fat diet and the Mediterranean diet, respectively (Fig 7B). Moreover, a second cluster of proteins (complement factor H-related protein, tetranectin, β-2-microglobulin, and apolipoprotein C-II) was identified as being up-regulated and down-regulated after the Mediterranean diet and the low-fat diet, respectively (Fig 7B).

#### Changes in the expression profiles of miRNAs in response to the diet according to the endothelial dysfunction severity

In patients with severe endothelial dysfunction, we observed lower levels of 5 miRNAs and higher levels of 24 miRNAs after the intervention with a Mediterranean diet compared with the low-fat diet (Table 4 and Fig 7C). Additionally, in patients with nonsevere endothelial dysfunction, we observed lower levels of 3 miRNAs and higher levels of 9 miRNAs after the follow-up period with a Mediterranean diet compared with the low-fat diet (Table 4 and Fig 7D).

We performed a bibliographic search and a mechanistic study into the role of miRNAs and their effect at proteomic level (S5 Fig). Patients who consumed the Mediterranean diet had lower *miR181c-5p* levels as compared with low-fat diet, inhibiting the proapoptotic action of this miRNA. It has been reported that *miR181c-5p* is also implicated in ROS synthesis, so the reduction of the levels of this miRNA would lead a decrease of GPx3, an enzyme that catalyzes the reduction of ROS, as we observed in our proteomic study [34]. We also found low levels of miRNA *let-7e-5p* after consumption of the Mediterranean diet compared with the low-fat diet. This miRNA has been reported to participate in the activation of nuclear factor kappa-light-chain-enhancer of activated B cells (NF-kB) and the consequent activation of the inflammatory pathway [35]. In this context, our proteomic analysis revealed a decrease in CRP (target gene of NF-kB) after the consumption of Mediterranean diet. Our results also suggest that the Mediterranean diet induced low levels of *miR-939-5p*, which regulate the expression of genes involved in proliferation and cellular migration (Stromal interaction molecule 1 [STIM1] and catenin) [36, 37], possibly increasing cell proliferation. Moreover, it has been reported that *miR-188* inhibits cyclin-dependent kinase 5 (CDK5) gene expression, which is a strong regulator of sirtuin 1 (SIRT1), which acts on cellular senescence and the development of atherosclerosis [38]. The increased levels of this miRNA, observed after the consumption of the Mediterranean diet, could explain the reduction of endothelial cell senescence. Finally, higher levels of *miR-25-5p*, an miRNA that inhibits a disintegrin and metalloproteinase domain-containing protein 10 (*Adam10*) gene expression involved in the activation of the inflammatory pathway induced by oxidized low-density lipoprotein (LDL), were observed after the Mediterranean diet (S5 Fig) [39].

**Table 4:** Changes in miRNA profile after dietary intervention in CHD patients classified according to the severity of endothelial dysfunction.

	Lower in Mediterranean Diet versus Low-Fat Diet	Higher in Mediterranean Diet versus Low-Fat Diet
**Nonsevere endothelial dysfunction (FMD >2%)**	*hsa-miR-181c-5p* *hsa-miR-325* *hsa-miR-877-5p*	*hsa-miR-1229-3p* *hsa-miR-188-5p* *hsa-miR-200b-5p* *hsa-miR-3131* *hsa-miR-3977* *hsa-miR-4468* *hsa-miR-4691-3p* *hsa-miR-653-3p* *hsa-miR-7154-5p*
**Severe endothelial dysfunction (FMD <2%)**	*hsa-let-7e-5p* *hsa-miR-4436a* *hsa-miR-485-3p* *hsa-miR-670-3p* *hsa-miR-939-5p*	*hsa-miR-1182* *hsa-miR-1287-5p* *hsa-miR-1292-5p* *hsa-miR-1587* *hsa-miR-1910-3p* *hsa-miR-202-5p* *hsa-miR-25-5p* *hsa-miR-3133* *hsa-miR-3136-5p* *hsa-miR-3650* *hsa-miR-3920* *hsa-miR-485-5p* *hsa-miR-5000-5p* *hsa-miR-5195-5p* *hsa-miR-532-3p* *hsa-miR-551a* *hsa-miR-6799-3p* *hsa-miR-6801-3p* *hsa-miR-6813-5p* *hsa-miR-6814-3p* *hsa-miR-6819-5p* *hsa-miR-7109-5p* *hsa-miR-7156-3p* *hsa-miR-874-3p*

**Abbreviations:** CHD, coronary heart disease; FMD, flow-mediated dilation; miRNA, micro-RNA.

## Discussion

Our results suggest that the consumption of 2 healthy dietary patterns exerted different effects on endothelial function and markers related to vascular endothelial homeostasis in CHD patients. In this sense, we observed a modulation of endothelial function after consumption of the Mediterranean diet, increasing FMD and EPC content and decreasing EMP levels and EMP/EPC ratio, regardless of the severity of endothelial dysfunction. Our data also suggest the Mediterranean diet decreased intracellular ROS production, cellular apoptosis, and endothelial cell senescence while increasing cellular proliferation and angiogenesis. Additionally, we observed a regulation of the levels of the miRNAs that are involved in the inhibition of genes and proteins related to biological processes directly associated with endothelial dysfunction and endothelial vascular homeostasis after consumption of this diet. However, the low-fat diet seems to have no effect on endothelial function in any group of patients, but we observed an increased endothelial damage regardless of the severity of endothelial dysfunction. Nevertheless, our data suggest that this diet slightly increased EPC content in patients with nonsevere endothelial dysfunction, although it failed in the capacity to increase EPCs in patients with severe endothelial dysfunction.

The notion that the Mediterranean diet produces an improvement in endothelial function has been widely described in different populations such as healthy elderly patients [20], metabolic syndrome patients [40], hypercholesterolemic patients [22, 41], or patients with type 2 diabetes mellitus [30]. The potential effect of the Mediterranean diet in improving endothelial dysfunction, as well as different cardiovascular risk factors (blood lipids, glucose, or inflammation), and reducing cardiovascular events (in primary and secondary prevention) [42, 43] could be attributed to its fatty acid profile, as well as to its high antioxidant content (mainly phenolic compounds such as hydroxytyrosol, tyrosol, phenyl alcohols, and flavonoids). In fact, our results suggest that the Mediterranean diet produced higher high-density lipoprotein (HDL) cholesterol levels and lower fasting glucose and high sensitive CRP (hsCRP) levels than the low-fat diet, in accordance with other dietary intervention studies [40, 44–46]. Moreover, in comparison to the low-fat diet, the Mediterranean diet provided a lower content of total carbohydrates, which suggests that this model of diet could also reduce the deleterious effect of carbohydrate-induced hyperglycemia on endothelial function [47]. This beneficial effect of the Mediterranean diet is also corroborated by the observed reduction of circulating MG levels, as we have described previously [48, 49]. MG—a major precursor of AGEs, which are generated endogenously by nonenzymatic glycation of proteins—can increase ROS production and cause oxidative stress by reacting within the vessel wall and modifying the structure and function of the endothelial vasculature [50].

We have previously shown that the short-term consumption (4 weeks) of a Mediterranean diet produced a higher number of EPCs and a lower EMP concentration, together with a greater ischemic reactive hyperemia, compared with both an SFA-rich diet and a low-fat, high-carbohydrate diet in an elderly population [51]. This potential relationship has also been described recently by Weech and colleagues [19], who showed a beneficial effect on endothelial repair and maintenance, increasing the number of EPCs and reducing EMP levels, by the replacement of dietary SFAs with MUFAs or n-6 PUFAs for 16 weeks in 190 patients at high risk of CVD. However, no association was found between EPC numbers and FMD, possibly because of the absence of phenolic compounds, since this study used refined olive oil as the primary source of MUFAs [19]. Taking these short-term study results into account, there remains a need to explore the effect of dietary models on endothelial vascular homeostasis for longer periods. We observed higher EPC content after the long-term consumption of the Mediterranean diet, even in those patients with severe endothelial dysfunction, suggesting that this dietary model provides additional benefits to endogenous repair mechanisms by counteracting EMP release and by replacing dysfunctional/damaged endothelium. Conversely, the low-fat diet seems to increase circulating EPC levels but only in those patients with nonsevere endothelial dysfunction (at lower risk of cardiovascular recurrence), exerting an imbalance of endothelial vascular homeostasis in patients with severe endothelial dysfunction. These results suggest that the Mediterranean diet, but not a low-fat diet, could be recommended as the best dietary strategy to recover severe endothelial dysfunction and therefore reduce the increased risk of presenting a cardiovascular recurrence in CHD patients with severe endothelial dysfunction.

In order to determine whether the diet could modulate cell mechanisms involved in endothelial vascular homeostasis associated with endothelial function impairment, we performed in vitro experiments on different endothelial cell models. We observed an increase in endothelial repair mechanisms (cellular proliferation and angiogenesis) and a decrease in mechanisms associated with endothelial damage (in vitro ROS production and cellular senescence and apoptosis) after the Mediterranean diet, regardless of the severity of endothelial dysfunction [52, 53]. In this way, the Mediterranean diet seem to induce changes in the levels of miRNAs that modulate the expression of specific target genes to regulate biological processes involved in endothelial dysfunction and vascular endothelial homeostasis, such as ROS synthesis, attenuation of cell senescence, promoting cellular migration and proliferation, and inhibition of the proinflammatory pathway. All these biological processes were reflected by increased levels of EPCs, low endothelial cell senescence, and a reduction of oxidative stress/inflammation.

Our study has a number of major strengths. It is well known that CHD patients who also have endothelial dysfunction are at high risk of recurrence of a cardiovascular event [12]. Therefore, our results provide a dietary strategy as a clinical and therapeutic tool that could reduce the high cardiovascular recurrence of these patients by improving endothelial dysfunction and achieving a better balance of endothelial vascular endothelial homeostasis by consuming a Mediterranean diet, but not a low-fat diet. Moreover, the study presents a randomized design that involves 2 dietary models involving a large number of patients (n = 805).

Nevertheless, the study also has its limitations. The evaluation of endothelial function is a secondary endpoint of the CORDIOPREV study. As in any substudy, the results of this study should be treated with caution. Secondly, because of the exploratory nature of the analyses, the possibility of false-positive findings could arise through multiple tests. Moreover, the results from a Mediterranean population may not be generalizable to other populations and require replication in other cohorts of coronary patients, such as those who have suffered a stroke. Genetic and environmental factors could also influence the prognostic value of FMD [54]. Finally, dietary compliance during such an extended period could be a factor, although in this case, adherence to the recommended dietary pattern was excellent, as shown by the rigorous dietary assessment measurements [28].

In summary, our data suggest that the consumption of a Mediterranean diet may improve endothelial dysfunction in CHD patients by achieving a better balance of vascular endothelial homeostasis, even in those at high risk of cardiovascular events (with severe endothelial dysfunction). We did not observe any effect on endothelial function, although it did seem to activate endothelial repair mechanisms, albeit only in patients with nonsevere endothelial dysfunction.

## Supporting information

S1 Text Material and methods. (DOC)

S1 Table Mean nutrient intake and dietary adherence of CHD patients^a^ at baseline and 1 year after randomization. ^a^One participant in the Mediterranean diet and one participant in the low-fat diet were excluded from calculations of food intake because their energy values were outside the prespecified ranges. Dietary assessment was conducted using a validated food frequency questionnaire (136 items). ^b^Based on the 14-item Mediterranean diet adherence screener. The range was 0 (minimum) to 14 (maximum) points. This tool was administered in 2 intervention groups. ^c^Based on a 9-item assessment questionnaire on adherence to a low-fat diet. The range was 0 (minimum) to 9 (maximum) points. This tool was only administered in the LFHCC diet as an internal control. *p < 0.05 (Student *t* test), participants in Mediterranean diet versus patients in the low-fat diet group. CHD, coronary heart disease; E, energy; LFHCC, low-fat diet rich in complex carbohydrates; MUFA, monounsaturated fatty acid; PUFA, polyunsaturated fatty acid; SFA, saturated fatty acid.
(DOCX)

S2 Table Adherence to each of the 14 items of the MEDAS (for the Mediterranean diet). Data are shown as percentage of participants with positive answer. *Statistically significant comparisons between the Mediterranean diet and the low-fat diet for each visit. Chi-squared tests were indicated with symbol *(*p* < 0.05). MEDAS, the 14-item MEditerranean Diet Adherence Screener; s/d, servings per day; s/w, servings per week.
(DOCX)

S3 Table Adherence to each of the 9 items of the low-fat diet screener. Values are expressed as percentage of participants giving positive answers. s/w, servings per week.
(DOCX)

S4 Table Food intake (grams/1,000 kcal) at baseline^a^ and after 1 year of dietary intervention by study group. (DOCX)

S5 Table Comparability of the 2 restricted groups across all baseline clinical parameters of the study. Values represent are means ± SE. *p < 0.05, total CORDIOPREV patients versus patients that completed follow-up ultrasound studies. BMI, body mass index; CORDIOPREV, CORonary Diet Intervention with Olive oil and cardiovascular PREVention; DBP, diastolic blood pressure; HDL, high-density lipoprotein; hsCRP, high sensitive C-reactive protein; LDL, low-density lipoprotein; SBP, systolic blood pressure.
(DOCX)

S6 Table Baseline characteristics of the CHD patients, selected for in vitro assays, according to FMD cutoff value. FMD < 2%: severe endothelial dysfunction; ≥2%: nonsevere endothelial dysfunction. Values represented are means ± SE. *p < 0.05 (one-way ANOVA), patients with FMD < 2% versus patients with FMD ≥ 2%. BMI, body mass index; CHD, cardiovascular heart disease; DBP, diastolic blood pressure; HDL, high-density lipoprotein; hsCRP, high sensitive C-reactive protein; LDL, low-density lipoprotein; SBP, systolic blood pressure.
(DOCX)

S1 Fig Effect of dietary intervention on endothelial function (measured by FMD) and markers of endothelial repair mechanisms and endothelial damage (circulating EPCs and EMPs) in CHD patients. CHD, coronary heart disease; EMP, endothelial microparticle; EPC, endothelial progenitor cell; FMD, flow-mediated dilation
(TIF)

S2 Fig Effect of dietary intervention (Δchanges produced between post- and preintervention) on in vitro intracellular ROS production (A, B, and C) and cellular apoptosis (D, E, and F) in HCAECs incubated with serum samples from 24 selected CHD patients. FMD < 2%, patients with severe endothelial dysfunction; FMD ≥ 2%, patients with nonsevere endothelial dysfunction. All data are mean ± SE. Continuous variables were compared using analysis of variance (univariate ANOVA). *Significant changes between Mediterranean diet and low-fat diet (*p* < 0.05). †Significant changes between post- and preintervention in each diet (*p* < 0.05). CHD, coronary heart disease; FMD, flow-mediated dilation; HCAEC, human coronary artery endothelial cell; ROS, reactive oxygen species.
(TIF)

S3 Fig Effect of dietary intervention (Δchanges produced between post- and preintervention) on in vitro cellular senescence (A, B, and C) and cellular proliferation (E, F, and G) in HCAECs incubated with serum samples from 24 selected CHD patients. (D) Representative optical microscopy images of in vitro senescence assay at final time point (24 h) (40×). FMD < 2%, patients with severe endothelial dysfunction; FMD ≥ 2%, patients with nonsevere endothelial dysfunction. All data are mean ± SE. Continuous variables were compared using analysis of variance (univariate ANOVA). †Significant changes between post- and preintervention in each diet (*p* < 0.05). *Significant changes between Mediterranean diet and low-fat diet (*p* < 0.05). CHD, coronary heart disease; FMD, flow-mediated dilation; HCAEC, human coronary artery endothelial cell; PCNA, proliferating cell nuclear antigen.
(TIF)

S4 Fig Evaluation of the MG levels after each dietary intervention (Δchanges produced between post- and preintervention) in the serum samples from 24 selected CHD patients. FMD < 2%, patients with severe endothelial dysfunction; FMD ≥ 2%, patients with nonsevere endothelial dysfunction. All data are mean ± SE. Continuous variables were compared using the analysis of variance (univariate ANOVA). *Significant changes between Mediterranean diet and low-fat diet (*p* < 0.05). †Significant changes between post- and preintervention in each diet (*p* < 0.05). CHD, coronary heart disease; FMD, flow-mediated dilation; MG, methylglyoxal.
(TIF)

S5 Fig Effect of the Mediterranean diet on the biological process associated with endothelial dysfunction and vascular endothelial homeostasis: from epigenetic to biochemical and proteomic factors. Results derived from a proteome screening by SWATH-MS analysis and expression profile of miRNAs by next-generation sequencing of serum samples from in vitro experiments. CRP, C-reactive protein; EPC, endothelial progenitor cells; GPx3, glutathione peroxidase 3; ox-LDL, oxidized low-density lipoprotein; ROS, reactive oxygen species.
(TIF)

## Abbreviations

ACEI: angiotensin-converting enzyme inhibitor
Adam10: a disintegrin and metalloproteinase domain-containing protein 10
AGE: advanced glycation end product
AHA: American Heart Association
CDK5: cyclin-dependent kinase 5
CHD: coronary heart disease
CI: confidence interval
CORDIOPREV: CORonary Diet Intervention with Olive oil and cardiovascular PREVention
CRP: C-reactive protein
CVD: cardiovascular disease
EMP: endothelial microparticle
EPC: endothelial progenitor cell
FMD: flow-mediated dilation
GPx3: glutathione peroxidase 3
HCAEC: human coronary artery endothelial cell
HDL: high-density lipoprotein
hsCRP: high sensitive CRP
HUVEC: human umbilical vein endothelial cell
LDL: low-density lipoprotein
MG: methylglyoxal
miRNA: micro-RNA
MUFA: monounsaturated fatty acid
NCEP: National Cholesterol Education Program
NF-kB: nuclear factor kappa-light-chain-enhancer of activated B cells
PUFA: polyunsaturated fatty acid
ROS: reactive oxygen species
ROTS: Reproducibility-Optimized Test Statistic
SFA: saturated fatty acid
SIRT1: sirtuin 1
STIM1: Stromal interaction molecule 1
