# Supplementary material for: Mediterranean diet and endothelial function in patients with coronary heart disease: An analysis of the CORDIOPREV randomized controlled trial
Source: PLoS Med. 2020 Sep 9;17(9):e1003282. doi: 10.1371/journal.pmed.1003282 (PMC7480872; doi:10.1371/journal.pmed.1003282)
Supplement: S1 Text — (DOC) [file pmed.1003282.s001.doc]

**Dietary intervention**

The dietary intervention was performed by a team of registered dietitians (RDs) who were previously trained to ensure uniformity and the quality of the intervention. The RDs conducted the dietary intervention with the same intensity in the two intervention groups.

At baseline and every 6 months, patients had an individual face-to-face visit with the RDs which included assessment of dietary intake and adherence, feedback, and reinforcement, as well as future directions. At each visit, RDs and patients worked together to identify dietary habits that needed to be changed, to set short-term goals and to work out how to make the changes. The achievements reached in the previous visits were used to increase patient motivation. Bimonthly telephone interviews were performed by the RDs to monitor compliance with the assigned diet, negotiate nutrition goals, and reinforce the dietary recommendations. In addition, group sessions of 20 participants were organized separately for each group every 3–4 months. These 2-h sessions included oral and written information (e.g., recipes, plans for meals, cooking tips, and shopping lists), group discussions, handouts, and reinforcement of dietary recommendations. To find social support, family members were encouraged to attend the individual and group sessions with the patient, especially if they shared the responsibility for food selection and the preparation of meals.

Written materials were designed and given to the patients at the individual and group sessions to enhance oral recommendations: leaflets summarizing the main food components and their frequency of consumption, and cooking recipes focused on increasing skills for preparing meals which complied with the assigned diet and meal plans. The patients also received free food to encourage dietary adherence: extra virgin olive oil rich in polyphenols in the Mediterranean diet group (approximately 1 L per week) and food packets containing low-fat products in the low-fat diet group.

**Dietary intake assessment**

Information on habitual dietary intake was collected at baseline and during follow-up using a 137-item semi-quantitative food-frequency questionnaire (FFQ), previously validated in the Spanish population [1]. Participants were asked to report their average intake of different

food and beverage items over the previous 12 months. For each item, typical portion size was included, and consumption frequencies were registered in nine categories ranging from “never or hardly ever” to “≥ six times/day”. As nutrient intake may vary in response to the availability of seasonal foods, the consumption of these foods was recorded for the

season and then adjusted by the proportional intake over 1 year. Energy and nutrient intake were calculated using the Spanish Food Composition Tables [2].

**Dietary adherence assessment**

The validated 14-item MEditerranean Diet Adherence Screener (MEDAS) was the method for assessing adherence to the Mediterranean diet used in our study [3]. This method was shown to be a valid tool in a large Spanish cohort for rapid estimation of adherence to the traditional Mediterranean diet [4]. As for assessing adherence to the low-fat diet, a 9-item dietary screener was used [5]. In both dietary screeners, a value of 0 (non-compliant) or 1 (compliant) was assigned to each item and higher scores reflected better adherence. Registered dietitians assessed the participants’ adherence using these tools during each visit. The MEDAS was performed in the two intervention groups, while the 9-item dietary screener was only run in the low-fat diet group (**S1, S2, S3 and S4 Tables**).

**Estimation of the sample size**

To calculate the sample size, we made the assumption that FMD in CHD patients is usually under 4.5% [6, 7]. Taking a two-tailed alpha value of 0.05, a follow-up loss rate of 2% (CORDIOPREV study loss rate) and a power error of 0.80, 145 subjects in each group were required to detect a minimum difference of 1 between both groups (according to our previous results [8]), assuming that there were two groups and a standard deviation of 3. Subsequently, given the fact that we used the CORDIOPREV population (1002 patients, of whom 805 completed the ultrasound follow-up study), the population size was considered suitable for our study.

**Laboratory Tests**:

At 8.00 am, following a 12-h fast, the patients were admitted to the laboratory for anthropometric and biochemical tests [BMI, waist circumference, systolic blood pressure, diastolic blood pressure, HDL-cholesterol, LDL-cholesterol, triglycerides, cholesterol, high sensitive C-reactive protein (hsCRP), glucose and HbA1c]. The patients had refrained from smoking during the fasting period and abstained from alcohol intake for the past 7 days. Venous blood was sampled from the antecubital vein and collected in Vacutainer tubes with no anticoagulant and then put into tubes containing EDTA, which were immediately transferred to 4 °C. The plasma and serum samples were frozen at −80 °C for further biochemical analysis. The serum parameters were measured in Architect c-16000 analyzers (Abbott, Chicago, IL, USA) using spectrophotometric techniques (enzymatic colorimetric methods): the hexokinase method for glucose and oxidation–peroxidation for cholesterol; the triglycerides, LDL-cholesterol and HDL-cholesterol levels were estimated using the Friedewald formula, based on cholesterol, triglycerides, and HDL-cholesterol concentrations. The plasma levels of insulin were measured by chemiluminescent microparticle immunoassay using an analyzer (i-2000Abbott Architect, Chicago, IL, USA). The plasma concentrations of hsCRP were determined by high sensitivity ELISA (BioCheck, Inc., Foster City, CA, USA).

**Quantification of EPCs**

Briefly, 100 μl of whole blood was incubated for 20 min at 4◦ C in darkness with 15 μl of TC (Tricolour)-conjugated anti-(human-CD34) antibody (Invitrogen) and FITC-conjugated anti-(human VEGF) antibody (R&D Systems) and PE-conjugated anti-(human-CD133) (Miltenyi Biotec). Isotype-identical antibodies served as controls (BD Pharmigen). Later, red blood cells and platelets were lysed with 1 mL of Versalyse (Beckman Coulter) and then vortexed and incubated for 30 min at room temperature in the dark. The remaining cells were analyzed by flow cytometry (Cytometer FC500; Beckman Coulter). CD34++VEGF++CD133++ cells were quantified and defined as EPCs. Each analysis included 150,000 events passing the gate [9, 10]. Our results are expressed as the proportion of positive cells for the three markers in relation to the total number of gated cells [11-13]. All data were analyzed using commercially available software (CXP analysis software 2.2; Beckman Coulter).

**Quantification of EMPs**

The isolation of microparticles (MPs) was performed, before and after 1 year of dietary intervention, following the procedures from Suades et al. [14]. After obtaining the total MPs from blood cells, EMPs were identified by their specific marker positivity, binding of annexin V and FSC/SSC characteristics, according to the methodology described previously [15, 16]. The identification and quantification of MPs were performed by flow cytometry in a FC500 cytometer (Beckman Coulter). The gate limits of the acquisition were previously adjusted, and the acquisition was performed for 1 min per sample. The data were then analyzed with Kaluza software (Beckman Coulter). The concentration (number of MPs per μL of PFP) was determined according to Nieuwland’s equation [16], which includes the sample volume of the sample, the flow rate of the cytometer and the number of fluorescence-positive events (N), MPs/ μL = N x (Vf/Va) x (Vt/FR) x (1/Vi), where, Vf (μL) = final volume of washed MP suspension, Va (μL) = volume of washed MP suspension used for each labelling analysis, Vt (μL) = total volume of MP suspension before fluorescence-activated cell sorting analysis, FR(μL/min) = flow rate of the cytometer in low mode (average volume of microparticle suspension analyzed in 1 min), 1 is the μL unit of volume, and Vi(μL) = original volume of plasma used for microparticle isolation. The quantity of MPs was expressed as the number of EMPs/μL of PFP.

**Cell culture studies in endothelial-related cells**

***- Estimation of the sample size for in vitro experiments***

Due the fact that there are no similar studies which analyze the effect of serum samples from CHD patients after a long-term period (1 year) of diet in an endothelial cell model, the sample size was calculated according to our previous results from *in vitro* measurements of intracellular ROS production in HUVECs in a healthy elderly population with a shorter dietary intervention period (4 weeks) [17]. Therefore, taking into account the higher expected ROS concentration and the greater effect of the current dietary intervention (1 year), the calculation of the sample size for our study was based on a minimum difference of 25 and a standard deviation of 15 in intracellular ROS production after each dietary intervention. Following these guidelines, a minimum of 6 patients/group is required.

***- Human umbilical vein endothelial cells*** (HUVEC; Cambrex Bio Science Walkersville, Inc) were cultivated until confluence was reached (3–4 days). Cell culture was performed in an endothelial growth medium (EGM) SingleQuots (Lonza Walkersville, Inc) containing 20% fetal calf serum (FCS, Lonza), in a humidified atmosphere (37°C, 5% CO2). The culture medium was changed every 2 or 3 days, and the cells were detached using trypsin-EDTA (Lonza Walkersville, Inc) [18].

***- Endothelial progenitor cells*** (EPCs derived and isolated from human umbilical venous blood, taken after informed consent was given by women in labor during cesarean sections). All the procedures followed were in accordance with the ethical standards of the institutional and national committees responsible for human experimentation and with the Helsinki Declaration of 1975. The blood mononuclear cell fraction (MNCs) was isolated from the buffy coats through density-gradient centrifugation with 20 ml Ficoll-Paque (Gibco-Invitrogen, Grand Island, NY). Centrifugation was carried out for 35 min at 400 x g. The interphase layer of MNC was carefully aspirated and washed in PBS containing 2 mM EDTA and further centrifuged for 10 min at 200 x g. The cell pellet was then resuspended in 300 μl buffer and cultured for further cell propagation. Next, the mononuclear cells were plated on fibronectin-coated 6-well culture plates (Sigma Chemical Co., St. Louis, MO) at a density of 5×106 cells per well and supplemented with 20 % FCS, penicillin (100 U/ml) and streptomycin (100 μg/ml). After 2 days, any non-adherent cells were collected and plated on fibronectin-coated 12-wells at a density of 1×106 cells per well. The medium was topped up every 2 days for a week [19]. To characterize the EPC phenotype, cells were detached using trypsin-EDTA (Lonza Walkersville, Inc). The EPCs could be visualized with an inverted fluorescent microscope in the form of colonies (colony forming units, CFUs) and FITC-labeled Ulex europaeus agglutinin 1 (UEA-1, Sigma Chemical Company) and were then quantified in a flow cytometer (FACSCalibur, Becton Dickinson, USA).

***- Human Coronary Artery Endothelial Cells*** (HCAEC; CC-2585, Lonza Walkersville, Inc) were cultivated until confluence was reached (5–9 days). Cell culture was performed in an endothelial growth medium EBM™-2 Medium (CC-3156, Lonza Walkersville, Inc) and EGM™-2 MV Microvascular Endothelial Cell Growth Medium SingleQuots™ supplements (CC-4147, Lonza Walkersville, Inc) containing 5% fetal bovine serum (FBS), in a humidified atmosphere (37°C, 5% CO2). The culture medium was changed every 2 days, and the cells were detached using trypsin-EDTA (Lonza Walkersville, Inc).

**Detection of intracellular ROS production**

Hydroethidine (Invitrogen, Molecular Probes, Eugene, OR, USA), a substance which is oxidized by ROS, turns into ethidium and emits a red marker, was used to measure superoxide anion. The HUVEC and HCAEC monolayers were incubated in EGM or EBM™-2, respectively, without FCS for 4 h at 37°C with CO_2_. The HUVECs and HCAECs were then exposed to EGM or EBM™-2, respectively, supplemented with serum samples (10%) from each CHD patient in the study (at baseline and 1 year after each dietary intervention) for another 4-h period at 37°C with CO2. At the end of the treatments, the cells were exposed for 15 min at 37°C to 2 μM hydroethidine. The analyses were performed on a flow cytometer (FACSCalibur, Becton Dickinson, USA). The intra-assay coefficient of variation was 8.8% for HUVECs and 9.3% for HCAECs. Intracellular ROS production was measured as a percentage of positive cells marked with hydroethidine.

**Cellular apoptosis: TUNEL assay**

HUVEC and HCAEC cellular apoptosis was measured using a commercial kit based on terminal deoxynucleotidyl transferase (TdT)-mediated dUTP nick-end labeling (TUNEL; In situ cell death detection kit, Roche Diagnostics, Mannheim, Germany). The HUVEC and HCAEC monolayers were incubated in EGM or EBM™-2, respectively, without FCS for 24 h at 37°C with 5% CO_2_. Then, the HUVECs and HCAECs were exposed to EGM or EBM™-2, respectively, supplemented with serum samples (10%) from each CHD patient in the study (at baseline and 1 year after each dietary intervention) for another 24 h period at 37°C with 5% CO2. HUVEC and HCAEC cellular apoptosis was analyzed as previously described [18]. The apoptotic index was evaluated by counting the number of cells showing TUNEL positivity over the total number of cells (100,000 cells). The intra-assay coefficient of variation was 7.9% for HUVECs and 8.6% for HCAECs.

**Proliferation assay**

Cell proliferation was measured by flow cytometry using a kit containing anti-PE–proliferating cell nuclear antigen (PCNA) antibody (BD Pharmingen, San Diego, CA, USA). The EPCs and HCAECs (10^6^/ml) were exposed to a medium containing serum samples (10%) from each CHD patient in the study (at baseline and 1 year after each dietary intervention) for a 24-h period at 37°C with 5% CO_2_. The cells were then washed, fixed, permeabilized and stained. After permeabilization, an anti-PCNA antibody or the corresponding isotype control was added. After incubation, the cells were washed and resuspended in 0.5 ml 1% formaldehyde and stored at 4ºC for flow cytometry analysis [20]. The intra-assay coefficient of variation was 9.1% for EPCs and 8.3% for HCAECs.

**Senescence assay**

The EPCs and HCAECs were cultured and exposed to a medium containing serum samples (10%) from each CHD patient in the study (at baseline and 1 year after each dietary intervention) for a 24 h period at 37°C with CO2. The cells were fixed at 70% confluence and then incubated at 37ºC overnight, following the recommendations of the Acidic β-Galactosidase Staining commercial kit (SA-β-gal Staining cat. no. CBA-230, Cell Biolabs, Inc., San Diego, CA). The cells were then observed under microscopy (Eclipse Ti-S, Nikon Instruments Europe B.V., Badhoevedorp, The Netherlands) and any development of blue color was noted [20]. The results were expressed as a percentage of β-gal positive cells (blue-stained cells). The intra-assay coefficient of variation was 10.2% for HUVECs and 9.7% for HCAECs.

**Angiogenesis assay in matrigel**

Angiogenesis experiments were carried out, in EPCs, using 15-well plates (μ-plates, IBIDI GmbH) coated with 10 μl of Matrigel (Corning) and incubated at 37 °C for 1 h. For the angiogenesis assay, a total of 2500 cells/well were plated and incubated with serum samples (10%) from each CHD patient in the study (at baseline and 1 year after each dietary intervention), as well as a VEGF internal positive control (50 ng/ml), for a period of 10 h, at 37°C with CO_2_ [21]. Once this period had elapsed, the formation of vascular structures was quantified using IMAGEj, taking six microphotographs (Eclipse Ti-S, Nikon Instruments Europe B.V., Badhoevedorp, The Netherlands) of triplicate wells. Two parameters were taken for the quantification experiments (total master segments length and master junction number). The intra-assay coefficient of variation was 11.4%.

**Study on the role of other factors associated with endothelial dysfunction and vascular endothelial homeostasis.**

***Quantification of serum level of methylglyoxal (MG)***

Methylglyoxal (MG) is one of the most commonly studied AGEs, and it serves as a marker of AGE accumulation in several tissues. Serum MG was determined by well-validated competitive ELISAs based on non-cross-reactive monoclonal antibodies (mabs) for protein-bound MG derivatives [lysine-MG-H1 (3D11 mab)], characterized by HPLC, and used as immunogens [22, 23]. The resulting values reflect relatively stable protein- or peptide-associated MG and not the free compounds. The Pearson correlation analysis was used to check the correlation between MG levels and the in vitro cell mechanisms studied (ROS production, cellular senescence, proliferation, apoptosis and angiogenesis).

***SWATH quantitative proteomics analysis***

Serum proteins were precipitated with TCA/acetone and solubilised in 50 µL of 0.2% RapiGest SF (Waters, Milford, MA, USA) in 50 mM ammonium bicarbonate. The total protein content was measured using the Qubit Protein Assay Kit (Thermo Fisher Scientific, Waltham, MA, USA) and 50 µg of protein was subjected to trypsin digestion following a protocol adapted from Vowinckel et al. [24]. SWATH quantitative proteomics analysis was performed following Ortea et al. [25]. Briefly, a spectral library was built using data-dependent acquisition LC-MS runs from the pooled samples using a Triple TOF 5600 (Sciex, Redwood City, CA, USA) mass spectrometer using a top 65 data-dependent acquisition method with a 90 min reversed-phase nano-LC gradient. These runs were searched for against a human SwissProt target-reverse decoy database containing 20,200 human protein sequences, using Protein Pilot software (v5.0.1, Sciex) with a 1% FDR threshold. The MS/MS spectra of the peptides which had been identified (confidence score > 99%) were then used to generate a spectral library using the MS/MSALL with SWATH Acquisition MicroApp (version 2.0, Sciex).

Each individual sample was analyzed (1 μg in column) using a SWATH data-independent acquisition LC-MS method, consisting of 50 variable m/z isolation windows optimized with the previous DDA runs. The mass spectrometer and nano-LC gradient was the same as for the DDA runs. All the runs were aligned using 39 peptides of the protein Complement C3, and fragment ion chromatogram traces were extracted from the SWATH runs using the MS/MSALL with SWATH Acquisition MicroApp (v2.0), selecting up to ten peptides per protein, seven fragments per peptide, 50 ppm width and a FDR below 1%. The protein quantitation values extracted were then normalized for inter-run variability using Marker View (v. 1.2.1, Sciex).

***Analysis of miRNA expression by next-generation sequencing in serum samples***

The profile expressions of 2,083 human miRNA were assessed in 21 pool samples, which included serum from patients in the conditions of the study. Thus, the EdgeSeq miRNA Whole Transcriptome Assay from HTG Molecular Diagnostics, Inc. (AZ, USA), based on next-generation sequencing (NGS), was used to measure miRNA expression. The procedure was carried out according to the requirements and standards used in the molecular biology laboratory of the Instituto Valenciano de Oncología (IVO). The assay version used in this study included 13 housekeeping (HK) mRNAs, four positive controls and five negative controls. Sample processing and library preparation and data normalization were conducted following the protocol of the HTG EdgeSeq miRNA Sample in the IVO.

**REFERENCES**

1. Martin-Moreno JM, Boyle P, Gorgojo L, Maisonneuve P, Fernandez-Rodriguez JC, Salvini S, et al. Development and validation of a food frequency questionnaire in Spain. Int J Epidemiol. 1993;22(3):512-9. Epub 1993/06/01. doi: 10.1093/ije/22.3.512. PubMed PMID: 8359969.

2. Fernandez-Ballart JD, Pinol JL, Zazpe I, Corella D, Carrasco P, Toledo E, et al. Relative validity of a semi-quantitative food-frequency questionnaire in an elderly Mediterranean population of Spain. Br J Nutr. 2010;103(12):1808-16. Epub 2010/01/28. doi: 10.1017/S0007114509993837. PubMed PMID: 20102675.

3. Martinez-Gonzalez MA, Garcia-Arellano A, Toledo E, Salas-Salvado J, Buil-Cosiales P, Corella D, et al. A 14-item Mediterranean diet assessment tool and obesity indexes among high-risk subjects: the PREDIMED trial. PLoS One. 2012;7(8):e43134. Epub 2012/08/21. doi: 10.1371/journal.pone.0043134. PubMed PMID: 22905215; PubMed Central PMCID: PMCPMC3419206.

4. Schroder H, Fito M, Estruch R, Martinez-Gonzalez MA, Corella D, Salas-Salvado J, et al. A short screener is valid for assessing Mediterranean diet adherence among older Spanish men and women. J Nutr. 2011;141(6):1140-5. Epub 2011/04/22. doi: 10.3945/jn.110.135566. PubMed PMID: 21508208.

5. Estruch R, Ros E, Martinez-Gonzalez MA. Mediterranean diet for primary prevention of cardiovascular disease. N Engl J Med. 2013;369(7):676-7. Epub 2013/08/16. doi: 10.1056/NEJMc1306659. PubMed PMID: 23944307.

6. Schroeder S, Enderle MD, Ossen R, Meisner C, Baumbach A, Pfohl M, et al. Noninvasive determination of endothelium-mediated vasodilation as a screening test for coronary artery disease: pilot study to assess the predictive value in comparison with angina pectoris, exercise electrocardiography, and myocardial perfusion imaging. Am Heart J. 1999;138(4 Pt 1):731-9. PubMed PMID: 10502220.

7. Enderle MD, Schroeder S, Ossen R, Meisner C, Baumbach A, Haering HU, et al. Comparison of peripheral endothelial dysfunction and intimal media thickness in patients with suspected coronary artery disease. Heart. 1998;80(4):349-54. PubMed PMID: 9875110; PubMed Central PMCID: PMCPMC1728800.

8. Torres-Pena JD, Garcia-Rios A, Delgado-Casado N, Gomez-Luna P, Alcala-Diaz JF, Yubero-Serrano EM, et al. Mediterranean diet improves endothelial function in patients with diabetes and prediabetes: A report from the CORDIOPREV study. Atherosclerosis. 2018;269:50-6. doi: 10.1016/j.atherosclerosis.2017.12.012. PubMed PMID: 29274507.

9. Fernandez JM, Rosado-Alvarez D, Da Silva Grigoletto ME, Rangel-Zuniga OA, Landaeta-Diaz LL, Caballero-Villarraso J, et al. Moderate-to-high-intensity training and a hypocaloric Mediterranean diet enhance endothelial progenitor cells and fitness in subjects with the metabolic syndrome. Clin Sci (Lond). 2012;123(6):361-73. doi: 10.1042/CS20110477. PubMed PMID: 22489903.

10. Khan SS, Solomon MA, McCoy JP, Jr. Detection of circulating endothelial cells and endothelial progenitor cells by flow cytometry. Cytometry B Clin Cytom. 2005;64(1):1-8. doi: 10.1002/cyto.b.20040. PubMed PMID: 15668988.

11. Marti-Fabregas J, Crespo J, Delgado-Mederos R, Martinez-Ramirez S, Pena E, Marin R, et al. Endothelial progenitor cells in acute ischemic stroke. Brain Behav. 2013;3(6):649-55. doi: 10.1002/brb3.175. PubMed PMID: 24363968; PubMed Central PMCID: PMCPMC3868170.

12. Hristov M, Schmitz S, Schuhmann C, Leyendecker T, von Hundelshausen P, Krotz F, et al. An optimized flow cytometry protocol for analysis of angiogenic monocytes and endothelial progenitor cells in peripheral blood. Cytometry A. 2009;75(10):848-53. doi: 10.1002/cyto.a.20772. PubMed PMID: 19739088.

13. Masouleh BK, Baraniskin A, Schmiegel W, Schroers R. Quantification of circulating endothelial progenitor cells in human peripheral blood: establishing a reliable flow cytometry protocol. J Immunol Methods. 2010;357(1-2):38-42. doi: 10.1016/j.jim.2010.03.015. PubMed PMID: 20381496.

14. Suades R, Padro T, Vilahur G, Badimon L. Circulating and platelet-derived microparticles in human blood enhance thrombosis on atherosclerotic plaques. Thromb Haemost. 2012;108(6):1208-19. doi: 10.1160/TH12-07-0486. PubMed PMID: 23138460.

15. Biro E, Akkerman JW, Hoek FJ, Gorter G, Pronk LM, Sturk A, et al. The phospholipid composition and cholesterol content of platelet-derived microparticles: a comparison with platelet membrane fractions. J Thromb Haemost. 2005;3(12):2754-63. doi: 10.1111/j.1538-7836.2005.01646.x. PubMed PMID: 16359513.

16. Suades R, Padro T, Alonso R, Mata P, Badimon L. Lipid-lowering therapy with statins reduces microparticle shedding from endothelium, platelets and inflammatory cells. Thromb Haemost. 2013;110(2):366-77. doi: 10.1160/TH13-03-0238. PubMed PMID: 23740299.

17. Marin C, Ramirez R, Delgado-Lista J, Yubero-Serrano EM, Perez-Martinez P, Carracedo J, et al. Mediterranean diet reduces endothelial damage and improves the regenerative capacity of endothelium. Am J Clin Nutr. 2011;93(2):267-74. doi: 10.3945/ajcn.110.006866. PubMed PMID: 21123460.

18. Marin C, Delgado-Lista J, Ramirez R, Carracedo J, Caballero J, Perez-Martinez P, et al. Mediterranean diet reduces senescence-associated stress in endothelial cells. Age (Dordr). 2012;34(6):1309-16. doi: 10.1007/s11357-011-9305-6. PubMed PMID: 21894446; PubMed Central PMCID: PMCPMC3528364.

19. Abd El Aziz MT, Abd El Nabi EA, Abd El Hamid M, Sabry D, Atta HM, Rahed LA, et al. Endothelial progenitor cells regenerate infracted myocardium with neovascularisation development. J Adv Res. 2015;6(2):133-44. doi: 10.1016/j.jare.2013.12.006. PubMed PMID: 25750747; PubMed Central PMCID: PMCPMC4348451.

20. Ramirez R, Carracedo J, Nogueras S, Buendia P, Merino A, Canadillas S, et al. Carbamylated darbepoetin derivative prevents endothelial progenitor cell damage with no effect on angiogenesis. J Mol Cell Cardiol. 2009;47(6):781-8. doi: 10.1016/j.yjmcc.2009.09.005. PubMed PMID: 19782086.

21. Luna C, Carmona A, Alique M, Carracedo J, Ramirez R. TNFalpha-Damaged-HUVECs Microparticles Modify Endothelial Progenitor Cell Functional Activity. Front Physiol. 2015;6:395. doi: 10.3389/fphys.2015.00395. PubMed PMID: 26733886; PubMed Central PMCID: PMCPMC4686689.

22. Lopez-Moreno J, Quintana-Navarro GM, Camargo A, Jimenez-Lucena R, Delgado-Lista J, Marin C, et al. Dietary fat quantity and quality modifies advanced glycation end products metabolism in patients with metabolic syndrome. Mol Nutr Food Res. 2017;61(8). Epub 2017/02/25. doi: 10.1002/mnfr.201601029. PubMed PMID: 28233454.

23. Lopez-Moreno J, Quintana-Navarro GM, Delgado-Lista J, Garcia-Rios A, Delgado-Casado N, Camargo A, et al. Mediterranean Diet Reduces Serum Advanced Glycation End Products and Increases Antioxidant Defenses in Elderly Adults: A Randomized Controlled Trial. J Am Geriatr Soc. 2016;64(4):901-4. Epub 2016/04/23. doi: 10.1111/jgs.14062. PubMed PMID: 27100598.

24. Vowinckel J, Capuano F, Campbell K, Deery MJ, Lilley KS, Ralser M. The beauty of being (label)-free: sample preparation methods for SWATH-MS and next-generation targeted proteomics. F1000Res. 2013;2:272. Epub 2013/01/01. doi: 10.12688/f1000research.2-272.v2. PubMed PMID: 24741437; PubMed Central PMCID: PMCPMC3983906.

25. Ortea I, Ruiz-Sanchez I, Canete R, Caballero-Villarraso J, Canete MD. Identification of candidate serum biomarkers of childhood-onset growth hormone deficiency using SWATH-MS and feature selection. J Proteomics. 2018;175:105-13. Epub 2018/01/11. doi: 10.1016/j.jprot.2018.01.003. PubMed PMID: 29317355.
