# Supplementary material for: Mediterranean diet and endothelial function in patients with coronary heart disease: An analysis of the CORDIOPREV randomized controlled trial
Source: PLoS Med. 2020 Sep 9;17(9):e1003282. doi: 10.1371/journal.pmed.1003282 (PMC7480872; doi:10.1371/journal.pmed.1003282)
Supplement: S1 Table — aOne participant in the Mediterranean diet and one participant in the low-fat diet were excluded from calculations of food intake because their energy values were outside the prespecified ranges. Dietary assessment was conducted using a validated food frequency questionnaire (136 items). bBased on the 14-item Mediterranean diet adherence screener. The range was 0 (minimum) to 14 (maximum) points. This tool was administered in 2 intervention groups. cBased on a 9-item assessment questionnaire on adherence to a low-fat diet. The range was 0 (minimum) to 9 (maximum) points. This tool was only administered in the LFHCC diet as an internal control. *p < 0.05 (Student t test), participants in Mediterranean diet versus patients in the low-fat diet group. CHD, coronary heart disease; E, energy; LFHCC, low-fat diet rich in complex carbohydrates; MUFA, monounsaturated fatty acid; PUFA, polyunsaturated fatty acid; SFA, saturated fatty acid. (DOCX) [file pmed.1003282.s002.docx]

**S1 Table.** Mean nutrient intake and dietary adherence of CHD patients^a^ at baseline and 1 year after randomization.

|  | **Baseline** | | | **1-year follow-up** | | |  |
| --- | --- | --- | --- | --- | --- | --- | --- |
|  | **Mediterranean diet**  (n=418) | **Low-fat diet**  (n=387) | *p* value* | **Mediterranean diet**  (n=418) | **Low-fat diet**  (n=387) | *p* value* |  |
| Energy (kcal/day) | 2234 ± 24 | 2239 ± 26 | 0.896 | 1947 ± 20 | 1797 ± 22 | <0.001 |  |
| Total protein (%E) | 18.5 ± 0.1 | 18.6 ± 0.1 | 0.769 | 17.8 ± 0.1 | 19.0 ± 0.2 | <0.001 |  |
| Total carbohydrates (%E) | 41.3 ± 0.3 | 41.7 ± 0.3 | 0.371 | 41.8 ± 0.3 | 45.0 ± 0.4 | <0.001 |  |
| Fiber (g/day) | 24.8 ± 0.4 | 25.0 ± 0.4 | 0.676 | 26.0 ± 0.4 | 24.9 ± 0.4 | 0.066 |  |
| Total fats (%E) | 37.3 ± 0.3 | 36.7 ± 0.3 | 0.102 | 37.5 ± 0.3 | 32.7 ± 0.3 | <0.001 |  |
| MUFA (%E) | 18.3 ± 0.2 | 17.9 ± 0.2 | 0.074 | 19.6 ± 0.2 | 14.7 ± 0.2 | <0.001 |  |
| PUFA (%E) | 6.4 ± 0.1 | 6.2 ± 0.1 | 0.237 | 6.5 ± 0.1 | 7.3 ± 0.2 | <0.001 |  |
| SFA (%E) | 8.9 ± 0.1 | 8.9 ± 0.1 | 0.821 | 7.8 ± 0.1 | 7.7 ± 0.1 | 0.293 |  |
| Cholesterol (mg/day) | 328.3 ± 4.7 | 327.7 ± 5.5 | 0.931 | 250.9 ± 3.8 | 258.8 ± 7.7 | 0.360 |  |
| 14-unit Mediterranean diet score ^b^ | 8.9 ± 0.1 | 8.7 ± 0.1 | 0.060 | 11.0 ± 0.09 | 7.7 ± 0.1 | <0.001 |  |
| 9-unit Low-fat diet score ^c^ | --- | 3.9 ± 0.1 |  | --- | 6.6 ± 0.1 |  |  |
| MUFA, Monounsaturated fatty acid; SFA, saturated fatty acid; PUFA, polyunsaturated fatty acid; E, energy  ^a^One participant in the Mediterranean diet and one participant in the Low-fat diet were excluded from calculations of food intake because their energy values were outside the pre-specified ranges. Dietary assessment was conducted using a validated food frequency questionnaire (136 items).  ^b^Based on the 14-item Mediterranean diet adherence screener. The range was 0 (minimum) to 14 (maximum) points. This tool was administered in two intervention groups.  ^c^Based on a 9-item assessment questionnaire on adherence to a low-fat diet. The range was 0 (minimum) to 9 (maximum) points. This tool was only administered in the LFHCC diet as an internal control.  *^*^* *p* < 0.05 (t-student), participants in Mediterranean diet vs patients in Low-fat diet. | | | | | | | |
