# Supplementary material for: Mediterranean diet and endothelial function in patients with coronary heart disease: An analysis of the CORDIOPREV randomized controlled trial
Source: PLoS Med. 2020 Sep 9;17(9):e1003282. doi: 10.1371/journal.pmed.1003282 (PMC7480872; doi:10.1371/journal.pmed.1003282)
Supplement: S2 Table — Data are shown as percentage of participants with positive answer. *Statistically significant comparisons between the Mediterranean diet and the low-fat diet for each visit. Chi-squared tests were indicated with symbol *(p < 0.05). MEDAS, the 14-item MEditerranean Diet Adherence Screener; s/d, servings per day; s/w, servings per week. (DOCX) [file pmed.1003282.s003.docx]

**S2 Table.** Adherence to each of the 14 items of the MEDAS (for the Mediterranean diet)

|  | | | | |
| --- | --- | --- | --- | --- |
|  | Baseline | | 1-year | |
|  | Low-fat diet  (n=387) | Mediterranean  diet (n=418) | Low-fat diet  (n=387) | Mediterranean  diet (n=418) |
| 1. Use of olive oil as main culinary fat | 99.0 | 97.1 | 60.2 | 99.0* |
| 2. Olive oil ≥4 tablespoons/day | 60.9 | 66.9 | 8.8 | 82.6* |
| 3. Vegetables ≥2 s/d | 78.0 | 77.9 | 79.6 | 86.7* |
| 4. Fruits ≥3 s/d | 55.0 | 56.7 | 61.0 | 76.7* |
| 5. Red or processed meats <1 s/d | 57.4 | 61.7 | 88.4 | 87.1 |
| 6. Butter, margarine, cream <1 s/d | 92.5 | 91.4 | 94.3 | 97.9* |
| 7. Sweet/carbonated beverages <1 s/d | 72.1 | 76.0 | 85.0 | 83.1 |
| 8. Wine ≥ 7 glasses/week | 26.4 | 29.0 | 27.4 | 37.1* |
| 9. Legumes ≥3 s/w | 37.0 | 42.4 | 41.9 | 65.2* |
| 10. Fish or seafood ≥3 s/w | 59.4 | 57.1 | 42.4 | 73.6* |
| 11. Commercial bakery products ≤2 s/w | 42.9 | 41.2 | 60.2 | 75.0* |
| 12. Tree nuts ≥3 s/w | 33.3 | 36.2 | 13.7 | 49.3* |
| 13. Poultry more than red meats | 72.4 | 76.7 | 88.1 | 92.1 |
| 14. Use of “sofrito” sauce ≥2 times/week | 84.0 | 86.7 | 16.8 | 96.2* |
| Data are shown as percentage of participants with positive answer. s/d, servings per day; s/w, servings per week. *Statistically-significant comparisons between the Mediterranean diet and the low-fat diet for each visit. (Chi-squared tests) were indicated with symbol *(p<0.05) | | | | |
