# Supplementary material for: Mediterranean diet and endothelial function in patients with coronary heart disease: An analysis of the CORDIOPREV randomized controlled trial
Source: PLoS Med. 2020 Sep 9;17(9):e1003282. doi: 10.1371/journal.pmed.1003282 (PMC7480872; doi:10.1371/journal.pmed.1003282)
Supplement: S3 Table — Values are expressed as percentage of participants giving positive answers. s/w, servings per week. (DOCX) [file pmed.1003282.s004.docx]

**S3 Table.** Adherence to each of the 9 items of the low-fat diet screener

|  | | | |
| --- | --- | --- | --- |
|  | Low-fat diet (n=387) | | |
| Items | Baseline | 1-year | *p* value |
| 1. Vegetable oils (≤2 tablespoons/day) | 2.6 | 47.3 | <0.001 |
| 2. Remove visible fat in meats and soups | 78.6 | 86.6 | 0.003 |
| 3. Red, processed fatty meats (≤1 s/w) | 11.4 | 63.8 | <0.001 |
| 4. Fatty spread (≤1 s/w) | 77.5 | 87.1 | <0.001 |
| 5. Low-fat dairy products | 52.2 | 71.8 | <0.001 |
| 6. Use of oil-free cooking techniques | 36.2 | 81.4 | <0.001 |
| 7. Fatty fish, seafood canned in oil (≤ 1 s/w) | 40.8 | 80.4 | <0.001 |
| 8. Commercial bakery products, sweets, pastries (≤ 1 s/w) | 37.2 | 59.2 | <0.001 |
| 9. Nuts and fried snacks (≤ 1 s/w) | 49.9 | 78.6 | <0.001 |
| Values are expressed as percentage of participants giving positive answers. s/w, servings per week. | | | |
