# Supplementary material for: Mediterranean diet and endothelial function in patients with coronary heart disease: An analysis of the CORDIOPREV randomized controlled trial
Source: PLoS Med. 2020 Sep 9;17(9):e1003282. doi: 10.1371/journal.pmed.1003282 (PMC7480872; doi:10.1371/journal.pmed.1003282)
Supplement: S4 Table — (DOCX) [file pmed.1003282.s005.docx]

| **S4 Table.** Food intake (grams/1000kcal) at baseline^a^ and after 1 year of dietary intervention by study group | | | | | | |
| --- | --- | --- | --- | --- | --- | --- |
|  | **Baseline** | | | **1-year follow-up** | | |
|  | **Mediterranean diet**  (n=418) | **Low-fat diet**  (n=387) | *p* value* | **Mediterranean diet**  (n=418) | **Low-fat diet**  (n=387) | *p* value* |
| Total grains (grams/1000kcal) | 77.4 ± 1.4 | 76.4 ± 1.4 | 0.331 | 70.5 ± 1.5 | 82.7 ± 1.7 | 0.001 |
| Whole grains (grams/1000kcal) | 18.0 ± 1.6 | 18.5 ± 1.7 | 0.823 | 32.9 ± 1.9 | 40.1 ± 1.9 | 0.045 |
| Refined grains (grams/1000kcal) | 59.4 ± 1.8 | 57.8 ± 2.0 | 0.582 | 37.6 ± 1.9 | 42.5 ± 2.0 | 0.068 |
| Vegetables (grams/1000kcal) | 120.0 ± 2.4 | 117.1 ± 2.6 | 0.412 | 146.6 ± 3.4 | 128.1 ± 2.6 | <0.001 |
| Fruit (grams/1000kcal) | 171. ± 4.8 | 168.5 ± 4.8 | 0.670 | 214.0 ± 5.3 | 219.9 ± 4.9 | 0.412 |
| Legumes (grams/1000kcal) | 10.2 ± 0.3 | 11.1 ± 0.4 | 0.074 | 13.4 ± 0.3 | 13.1 ± 0.5 | 0.882 |
| Nuts (grams/1000kcal) | 4.1 ± 0.9 | 3.7 ± 0.2 | 0.199 | 5.6 ± 0.3 | 2.3 ± 0.2 | <0.001 |
| Extra virgin olive oil (grams/1000kcal) | 13.9 ± 0.4 | 13.5 ± 0.4 | 0.060 | 20.6 ± 0.3 | 10.3 ±0.3 | <0.001 |
| Oily fish (grams/1000kcal) | 16.8 ± 0.5 | 16.2 ± 0.6 | 0.323 | 17.4 ± 0.5 | 13.8 ± 0.5 | <0.001 |
| Wine (grams/1000kcal) | 24.5 ± 1.8 | 24.9 ± 2.1 | 0.869 | 30.2 ± 2.3 | 29.1 ± 1.9 | 0.731 |
| Red meat/processed meats (grams/1000kcal) | 36.9 ± 0.8 | 38.9 ± 0.9 | 0.110 | 23.7 ± 0.7 | 30.4 ± 0.9 | <0.001 |
| Dairy products (grams/1000kcal) | 174.6 ± 4.3 | 162.9 ± 4.5 | 0.062 | 188.2 ± 5.3 | 209.0 ± 6.1 | 0.010 |
| Pastries/commercial bakery (grams/1000kcal) | 11.7 ± 0.5 | 11.9 ± 0.5 | 0.756 | 7.9 ± 0.4 | 12.9 ± 0.7 | <0.001 |
| Data are mean ± SEM. Data are presented as grams of food per 1000 kcal to facilitate the comparison between groups and between visits considering that there are differences in energy intake.  ^a^ One participant in the Mediterranean diet and one participant in the Low-fat diet were excluded from calculations of food intake because their energy values were outside the pre-specified ranges.  *^*^* *p* < 0.05 (t-student), participants in Mediterranean diet versus patients in Low-fat diet. | | | | | | |
