# Supplementary material for: Mediterranean diet and endothelial function in patients with coronary heart disease: An analysis of the CORDIOPREV randomized controlled trial
Source: PLoS Med. 2020 Sep 9;17(9):e1003282. doi: 10.1371/journal.pmed.1003282 (PMC7480872; doi:10.1371/journal.pmed.1003282)
Supplement: S5 Table — Values represent are means ± SE. *p < 0.05, total CORDIOPREV patients versus patients that completed follow-up ultrasound studies. BMI, body mass index; CORDIOPREV, CORonary Diet Intervention with Olive oil and cardiovascular PREVention; DBP, diastolic blood pressure; HDL, high-density lipoprotein; hsCRP, high sensitive C-reactive protein; LDL, low-density lipoprotein; SBP, systolic blood pressure. (DOCX) [file pmed.1003282.s006.docx]

**S5 Table.** Comparability of the two restricted groups across all baseline clinical parameters of the study*^1^*

|  | Patients that completed  follow-up ultrasound studies (n = 805) | | Total number of CORDIOPREV patients  (n = 1002) | *p* value* |
| --- | --- | --- | --- | --- |
|  |  | |  |  |
| Age (years) | 59.8 ± 0.3 | 59.6 ± 0.3 | | *N.S.* |
| Men/Women | 680/125 | 837/165 | |  |
| Weight (kg) | 84.5 ± 0.4 | 85.1 ± 0.4 | | *N.S.* |
| BMI (kg/m^2^) | 31.0 ± 0.3 | 31.1 ± 0.1 | | *N.S.* |
| Waist circumference (mm) | 104.8 ± 0.3 | 105.1 ± 0.3 | | *N.S.* |
| DBP (mmHg) | 77.2 ± 0.3 | 77.2 ± 0.4 | | *N.S.* |
| SBP (mmHg) | 138.7 ± 1.2 | 138.8 ± 0.7 | | *N.S.* |
| LDL-cholesterol (mg/dL) | 88.5 ± 1.7 | 86.0 ± 1.1 | | *N.S.* |
| HDL-cholesterol (mg/dL) | 41.8 ± 0.6 | 42.2 ± 0.4 | | *N.S.* |
| Total cholesterol (mg/dL) | 158.3 ± 2.1 | 159.0 ± 1.2 | | *N.S.* |
| Triglycerides (mg/dL) | 137.2 ± 2.3 | 137.2 ± 2.6 | | *N.S.* |
| Fasting glucose (mg/dL) | 112.4 ± 1.2 | 113.1 ± 2.6 | | *N.S.* |
| Fasting insulin (mU/L) | 10.9 ± 0.8 | 10.8 ± 0.5 | | *N.S.* |
| hsCRP (mg/mL) | 3.01 ± 0.11 | 3.10 ± 0.13 | | *N.S.* |
| Alcohol drinkers (%) | 22.84 | 20.43 | | *N.S.* |
| Smoking (%) | 11.38 | 9.25 | | *N.S.* |

*^1^* Values represent are means ± SE. BMI, body mass index; DBP, Diastolic blood pressure; SBP, Systolic blood pressure; LDL, low density lipoprotein; HDL, high-density lipoprotein; hsCRP, high sensitive C-reactive protein.

*^*^* *p* < 0.05, Total CORDIOPREV patients vs Patients that completed follow-up ultrasound studies
