# Supplementary material for: Mediterranean diet and endothelial function in patients with coronary heart disease: An analysis of the CORDIOPREV randomized controlled trial
Source: PLoS Med. 2020 Sep 9;17(9):e1003282. doi: 10.1371/journal.pmed.1003282 (PMC7480872; doi:10.1371/journal.pmed.1003282)
Supplement: S6 Table — FMD < 2%: severe endothelial dysfunction; ≥2%: nonsevere endothelial dysfunction. Values represented are means ± SE. *p < 0.05 (one-way ANOVA), patients with FMD < 2% versus patients with FMD ≥ 2%. BMI, body mass index; CHD, cardiovascular heart disease; DBP, diastolic blood pressure; HDL, high-density lipoprotein; hsCRP, high sensitive C-reactive protein; LDL, low-density lipoprotein; SBP, systolic blood pressure. (DOCX) [file pmed.1003282.s007.docx]

**S6 Table.** Baseline characteristics of the CHD patients, selected for *in vitro* assays, according to FMD cut-off value*^1,2^*

|  | Severe endothelial dysfunction  FMD < 2% (n = 12) | | Non-severe endothelial dysfunction  FMD ≥ 2% (n = 12) | *p* value* |
| --- | --- | --- | --- | --- |
|  |  | |  |  |
| Age (years) | 60.3 ± 0.8 | 60.1 ± 0.9 | | 0.601 |
| Men/Women | 10/2 | 11/1 | | 0.456 |
| Weight (kg) | 80.5 ± 1.1 | 85.9 ± 0.9 | | 0.187 |
| BMI (kg/m^2^) | 29.1 ± 0.3 | 29.5 ± 0.2 | | 0.811 |
| Waist circumference (mm) | 103.8 ± 1.0 | 104.1 ± 0.9 | | 0.889 |
| DBP (mmHg) | 79.1 ± 0.9 | 79.5 ± 0.8 | | 0.954 |
| SBP (mmHg) | 145.2 ± 3.2 | 139.5 ± 2.5 | | 0.208 |
| Circulating EPCs (%) | 2.33 ± 0.15 | 4.22 ± 0.19 | | 0.015 |
| EMPs/μL | 3248 ± 81 | 2532 ± 59 | | 0.021 |
| EMPs:EPCs ratio | 3.89 ± 0.25 | 2.37 ± 0.11 | | 0.019 |
| LDL-cholesterol (mg/dL) | 90.9 ± 1.3 | 82.0 ± 1.8 | | 0.012 |
| HDL-cholesterol (mg/dL) | 37.0 ± 1.1 | 39.7 ± 1.0 | | 0.752 |
| Total cholesterol (mg/dL) | 160.1 ± 2.9 | 144.6 ± 1.8 | | 0.022 |
| Triglycerides (mg/dL) | 130.6 ± 3.1 | 115.7 ± 2.0 | | 0.175 |
| Fasting glucose (mg/dL) | 114.9 ± 3.2 | 107.1 ± 1.6 | | 0.658 |
| Fasting insulin (mU/L) | 14.2 ± 1.2 | 12.6 ± 0.9 | | 0.726 |
| hsCRP (mg/mL) | 3.28 ± 0.31 | 2.21 ± 0.43 | | 0.105 |
| Alcohol drinkers (%) | 29.01 | 25.99 | | 0.444 |
| Smoking (%) | 13.98 | 10.03 | | 0.401 |

*^1^* FMD (Flow-mediated dilation of the brachial artery) <2%: Severe endothelial dysfunction; ≥2%: non-severe endothelial dysfunction

*^2^* Values represented are means ± SE. CHD, cardiovascular heart disease; BMI, body mass index; DBP, Diastolic blood pressure; SBP, Systolic blood pressure; LDL, low-density lipoprotein; HDL, high-density lipoprotein; hsCRP, high sensitive C-reactive protein.

*^*^* *p* < 0.05 (One-way ANOVA), patients with FMD <2% vs patients with FMD ≥ 2%
