# Supplementary figures and images for: Mediterranean diet and endothelial function in patients with coronary heart disease: An analysis of the CORDIOPREV randomized controlled trial
Source: PLoS Med. 2020 Sep 9;17(9):e1003282. doi: 10.1371/journal.pmed.1003282 (PMC7480872; doi:10.1371/journal.pmed.1003282)

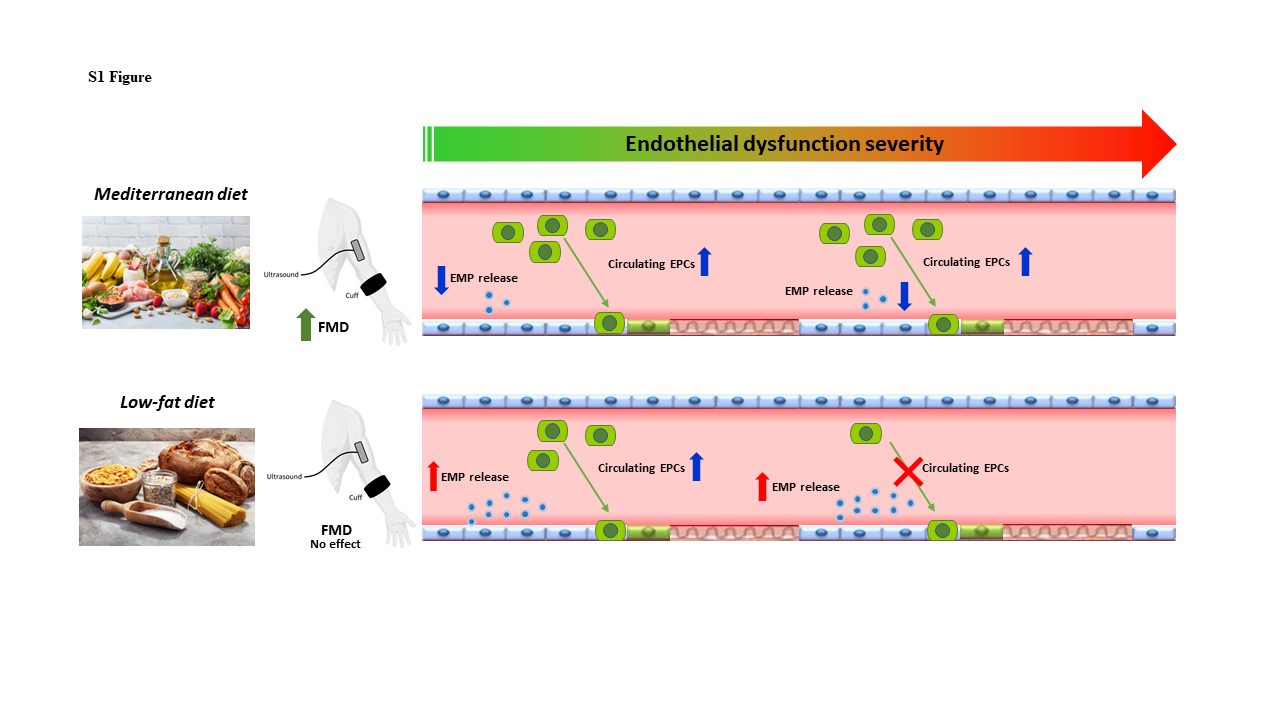

Supplement: S1 Fig — CHD, coronary heart disease; EMP, endothelial microparticle; EPC, endothelial progenitor cell; FMD, flow-mediated dilation (TIF) [file pmed.1003282.s008.tif]

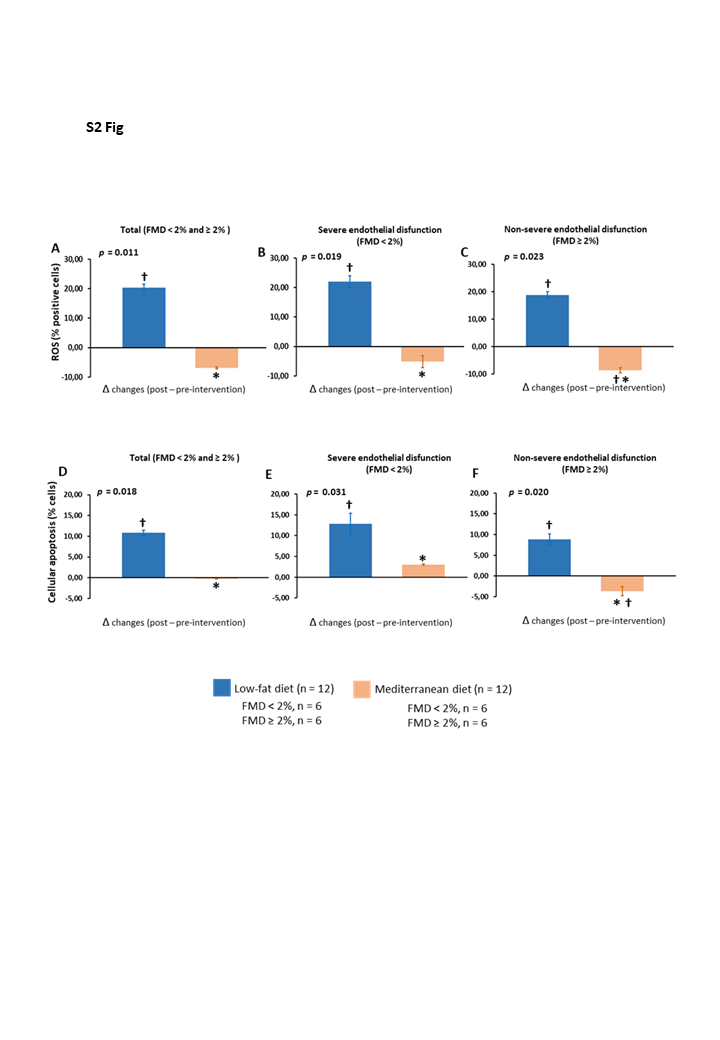

Supplement: S2 Fig — FMD < 2%, patients with severe endothelial dysfunction; FMD ≥ 2%, patients with nonsevere endothelial dysfunction. All data are mean ± SE. Continuous variables were compared using analysis of variance (univariate ANOVA). *Significant changes between Mediterranean diet and low-fat diet (p < 0.05). †Significant changes between post- and preintervention in each diet (p < 0.05). CHD, coronary heart disease; FMD, flow-mediated dilation; HCAEC, human coronary artery endothelial cell; ROS, reactive oxygen species. (TIF) [file pmed.1003282.s009.tif]

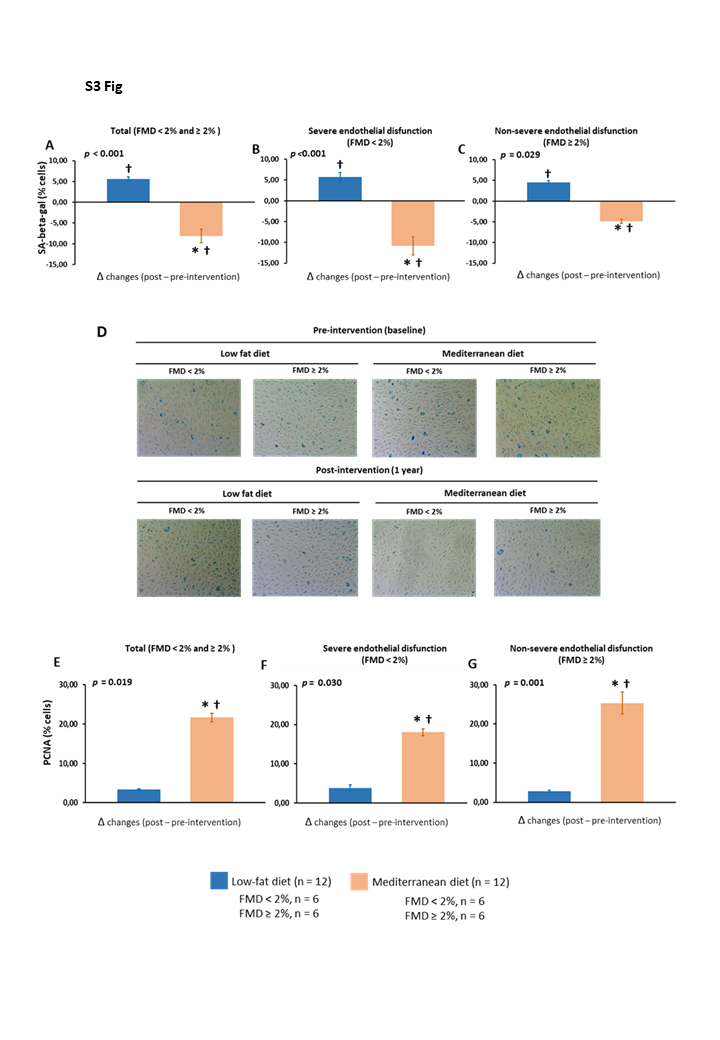

Supplement: S3 Fig — (D) Representative optical microscopy images of in vitro senescence assay at final time point (24 h) (40×). FMD < 2%, patients with severe endothelial dysfunction; FMD ≥ 2%, patients with nonsevere endothelial dysfunction. All data are mean ± SE. Continuous variables were compared using analysis of variance (univariate ANOVA). †Significant changes between post- and preintervention in each diet (p < 0.05). *Significant changes between Mediterranean diet and low-fat diet (p < 0.05). CHD, coronary heart disease; FMD, flow-mediated dilation; HCAEC, human coronary artery endothelial cell; PCNA, proliferating cell nuclear antigen. (TIF) [file pmed.1003282.s010.tif]

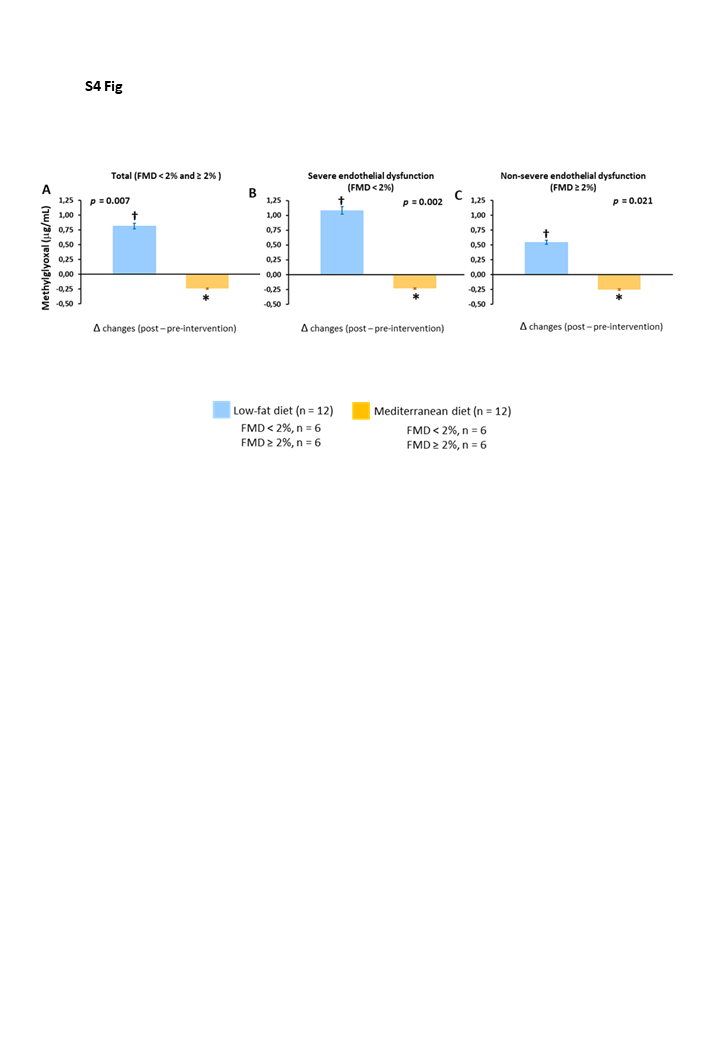

Supplement: S4 Fig — FMD < 2%, patients with severe endothelial dysfunction; FMD ≥ 2%, patients with nonsevere endothelial dysfunction. All data are mean ± SE. Continuous variables were compared using the analysis of variance (univariate ANOVA). *Significant changes between Mediterranean diet and low-fat diet (p < 0.05). †Significant changes between post- and preintervention in each diet (p < 0.05). CHD, coronary heart disease; FMD, flow-mediated dilation; MG, methylglyoxal. (TIF) [file pmed.1003282.s011.tif]

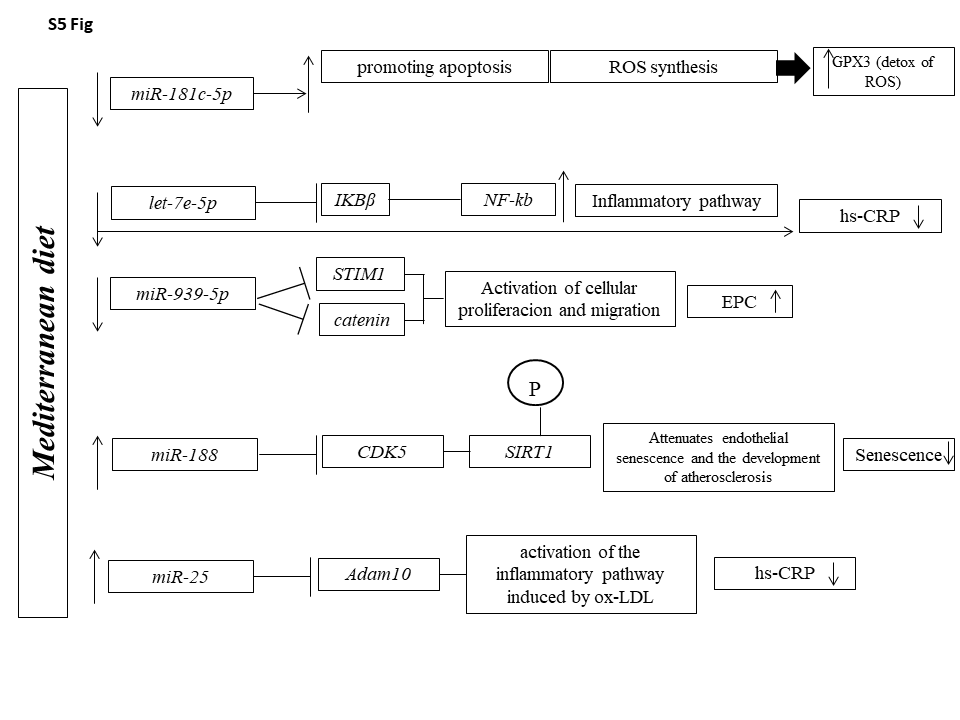

Supplement: S5 Fig — Results derived from a proteome screening by SWATH-MS analysis and expression profile of miRNAs by next-generation sequencing of serum samples from in vitro experiments. CRP, C-reactive protein; EPC, endothelial progenitor cells; GPx3, glutathione peroxidase 3; ox-LDL, oxidized low-density lipoprotein; ROS, reactive oxygen species. (TIF) [file pmed.1003282.s012.tif]
